# The modulation of calcium and chloride channels induces cardiomyocytes from human pluripotent stem cells

**DOI:** 10.7150/ijbs.95568

**Published:** 2025-01-01

**Authors:** Ya Meng, Chunhao Deng, Xia Xiao, Shengnan Wei, Chengcheng Song, Jiaxian Wang, Chon Lok Lei, Weiwei Liu, Guokai Chen

**Affiliations:** 1 Guangdong Provincial Key Laboratory of Tumor Interventional Diagnosis and Treatment, Zhuhai People's Hospital, Zhuhai Clinical Medical College of Jinan University, Zhuhai, Guangdong, 519000, China.; 2 Faculty of Health Sciences, University of Macau, Taipa, Macau.; 3 Chinese Medicine and Translational Medicine R&D center, Zhuhai UM Science & Technology Research Institute, Zhuhai, Guangdong, 519031, China.; 4 Biological Imaging and Stem Cell Core, Faculty of Health Sciences, University of Macau, Taipa, Macau.; 5 HELP Stem Cell Innovations Co. Ltd., Nanjing, 210000, China.; 6 MoE Frontiers Science Center for Precision Oncology, University of Macau, Macau SAR, China.

**Keywords:** calcium channel, ryanodine receptor 2, chloride channel, cardiomyocyte derivation, human pluripotent stem cells

## Abstract

Ion channels play a crucial role in cardiac functions, and their activities exhibit dynamic changes during heart development. However, the precise function of ion channels in human heart development remains elusive. In this study, we utilized human embryonic stem cells (hESCs) as a model to mimic the process of human embryonic heart development. During hESCs differentiation into cardiomyocytes, we observed differential expression of ion channel genes, including upregulation of ryanodine receptor 2 (RYR2), which encodes a calcium release channel. Subsequently, we discovered that Suramin, an activator of RyR2, efficiently promoted cardiac differentiation even in the absence of conventional WNT inhibitors. Furthermore, various modulators targeting sodium channels, potassium channels or chloride channels were examined under chemically defined conditions during cardiac differentiation. We found that DIDS, a chloride transport inhibitor, also enhanced hESCs differentiation into cardiomyocytes. Both Suramin and DIDS partially inhibited WNT signaling pathway, and RYR2 knockdown attenuated cardiac differentiation induced by WNT inhibitor treatment, or Suramin or DIDS administration. The resulting cardiomyocytes induced by these ion modulators exhibited specific expression patterns of cardiac genes and displayed typical electrophysiological signals. Notably, compared to WNT inhibitor treatment group, both Suramin and DIDS led to increased generation of atrial-like cardiomyocytes suggesting their potential as alternative inducers for specific cardiomyocyte lineage commitment during human cardiomyocyte induction processes. This study demonstrates that regulation of ion channels plays a crucial role in determining the fate of cardiac cells, providing an effective approach for inducing cardiomyocytes from hPSCs and highlighting their critical involvement in human heart development.

## Introduction

The generation of cardiac electrical impulse relies on the normal expression and function of ion channels located on the membrane of cardiomyocytes 1, 2. The activity of ion channels undergoes developmental changes during murine embryonic heart development 1, 2. Moreover, mutations and abnormal expressions of genes encoding ion channels have been associated with human congenital heart defects (CHD) 3, 4, indicating their involvement in normal heart development. However, due to the limitations in human embryo research, the precise function and molecular mechanism of ion channels in human heart development remain elusive.

Human embryonic stem cells (hESCs) possess the capacity to differentiate into various cell types within the human body, thereby providing a convenient platform for comprehending the regulatory network involved in human embryonic heart development. WNT pathway assumes a pivotal role in determining cardiac cell fate 5, 6. Generally, activation of the WNT pathway induces mesoderm lineage specification, followed by subsequent determination of cardiomyocyte cell fate through inhibition of WNT pathways 7-9. Our previous study reported that cardiomyocytes can be generated from hESC without requiring WNT inhibition 10, 11, suggesting potential involvement of alternative signaling pathways in cardiac cell fate determination.

Ion channels play a crucial role in cardiac and embryonic development in mice. Deletion of the sodium-calcium exchanger (*Ncx1*) in mice resulted in the lack of heartbeat, and retarded embryo development 12. Mutant mice lacking ryanodine receptor 2 (*Ryr2*) developed abnormal heart tube, and died at embryonic day 10 13. Furthermore, ion channels regulate cardiomyogenesis of mouse embryonic stem cells (mESCs). Inhibition of L-type calcium channel by nifedipine suppresses cardiac lineage commitment in mESCs 14. Activation of Ca^2+^-activated potassium channel by 1-ethyl-2-benzimidazolinone (EBIO) drives mESCs toward cardiomyocytes 15. Therefore, we hypothesize that ion channels are implicated in human heart embryonic development.

To elucidate the impact of ion channels on cardiac fate determination of human pluripotent stem cells (hPSCs), we conducted a comprehensive analysis of ion channel gene expression and subjected a panel of ion channel modulators during cardiac differentiation. Notably, we identified one RyR activator and two chloride channel modulators that effectively promoted cardiomyocyte differentiation in the absence of conventional WNT inhibitors. The resulting cardiomyocytes exhibited robust expression of cardiac marker genes, and displayed characteristic electrophysiological profiles. This study presents a highly efficient approach for generating functional cardiomyocytes from hPSCs, underscoring the pivotal role played by ion channels in directing hPSC lineage commitment toward the cardiac lineage.

## Materials and Methods

### Cell culture

H1 and H9 hESCs, and NL-4 hiPSCs were used in this study. The majority of experiments were conducted with the H1 line unless otherwise specified. hESCs and hiPSCs were maintained in E8 medium on Matrigel-coated plates (Corning). Cells are passaged with DPBS/EDTA in the presence of Y-27632 (Selleck) as previously described 16.

### Cardiomyocyte differentiation procedure

H1 and H9 hESCs, and NL-4 hiPSCs were maintained in E8 medium. When the cell density reached 80~90% (2-3 days after passaging), the medium was changed to differentiation medium (E5 basal medium: DMEM/F12, L-ascorbic acid, selenium, transferrin, Chemically Defined Lipid Concentrate and penicillin/streptomycin). CHIR99021 (5 μM) was added during day 0 in differentiation medium. IWP-2 (3 μM) or IWR1 (5 μM) was added from day 2 to day 5 as positive control of cardiac differentiation. Other inducers for cardiac differentiation were also added from day 2 to day 5, unless otherwise specified. The concentrations of ion channel modulators were listed in Supplementary Table 1. From day 2 to day 7, the cells were placed in E5 basal medium, and medium was changed every day. Insulin was added in the E5 basal medium from day 7 of differentiation. At the indicated time, the cells were harvested for further analyses, including quantitative real-time PCR (Q-PCR), RNA-seq, immunostaining or electrophysiology analysis.

H1 cells were treated with CHIR99021 for 1 day for WNT activation, and then supplemented IWP-2 (3 μM), suramin (50 μM) or DIDS (4 μM) for 24 hours to determine the influence of suramin or DIDS on WNT pathway.

### Immunostaining

To detect the presence and protein abundance of NKX2-5, TNNT2, and MLC2A, H1 hESCs were treated following the differentiation procedure. Then the cells were dissociated and re-plated onto coverslips coated with matrigel. The cells were fixed with 4% paraformaldehyde (PFA) for 10 minutes on ice, and then permeabilized with 0.1% Triton X-100 with 0.5% BSA for 15 minutes. Subsequently, the cells were incubated with primary antibodies overnight at 4 ℃. The primary antibodies used were NKX2-5 (sc-14033, Santa cruz), CT3 (TNNT2 antibody, DSHB) and MLC2A (311 011, Synaptic Systems). On the following day, the cells were washed with PBS and incubated with secondary antibodies (711-585-152 for NKX2-5 and 115-545-071 for CT3 and MLC2A) from Jackson ImmunoResearch Lab at room temperature (RT) for one hour, followed by staining with DAPI for 10 minutes in the dark. Confocal microscopy images were obtained with Carl Zeiss Confocal LSM710 or Leica TCS SP8.

### Flow cytometry

The cells were dissociated with TrypLE after 12 days of differentiation, followed by fixation with 1% PFA in PBS for 10 minutes. Then the cells were washed with PBS and permeabilized with 0.1% Triton X-100 in PBS for 10 minutes. Primary antibody anti CTNT (CT3, DSHB) was incubated with cells (1:100) for 2 hours. After washing twice, the cells were incubated with secondary antibody (Alexa Fluor® 488 conjugated, 115-545-071, Jackson ImmunoResearch Lab, Inc) for 1 hour. After washing, resuspended cells in PBS were subjected to flow cytometry analyses using a BD Accuri C6 flow cytometer.

### Quantitative real-time PCR

At day 10 of differentiation, the cells were harvested, and total RNA was extracted using RNAiso plus reagent (Takara) according to the manufacturer's protocol. 500 ng isolated RNA was reversed transcribed into cDNA using High-Capacity cDNA Reverse Transcription Kit (SuperScript III kit) from ThermoFisher. Takara SYBR® Premix Ex TaqTM II was applied to perform quantitative real-time PCR (qPCR) on Applied Biosystems QuantStudio 7 Flex. The data were obtained from three independent replicates and normalized using TBP. The relative mRNA levels were determined by dividing the normalized data by the normalized number of the control sample (the first sample shown in each bar chart).

### Next generation sequencing

At day 10 of differentiation, the cells were harvested, and total RNA was extracted with RNAiso plus. The sequencing library was generated using NEBNext® UltraTM RNA Library Prep Kit for Illumina® (NEB) following the manufacturer's guidance. Briefly, mRNA was purified from total RNA using poly-T oligo-attached magnetic beads. Then the index codes were added to each sample. Fragmentation of mRNA was performed in divalent cations in NEBNext First Strand Synthesis Reaction Buffer. First and second strand cDNA were synthesis using random hexamer primer and M-MuLV Reverse Transcriptase and DNA Polymerase I and RNase H, respectively. Subsequently, the overhangs were blunted via exonuclease/polymerase activities. Then, NEBNext Adaptor with hairpin loop were ligated. The library fragments were purified using AMPure XP system (Beckman Coulter) to select fragments of 20~300 base pairs (bp) in length. PCR was carried out using Phusion High-Fidelity DNA polymerase, Universal PCR primers and Index (X) Primer. The PCR products were purified with AMPure XP system and the library quality was assessed using the Agilent Bioanalyzer 2100 system. The library preparations were sequenced on an Illumina Hiseq platform according to the manufacturer's instructions and 125/150 bp paired-end reads were generated.

### RNA-seq data analysis

After sequencing, the raw reads in fastq format were processed by the Novogene Company. To obtain the clean data, reads with adaptors or ploy-N and low-quality reads were removed. All the downstream analyses were based on the clean data. Index of the reference human genome was built, and paired-end reads were aligned to the reference genome using Hisat2. Featurecount was employed to generate the read count and the TPM (transcripts per million). The differentially expressed genes between two samples were identified by edgeR, and a fold change of counts was no less than 1.5. Heatmaps were created using the pheatmap package in R, and hierarchical clustering was performed with similarity calculated by euclidean metric and complete linkage clustering. Functional enrichment of differentially expressed genes were performed using GO term and KEGG pathway analyses on the DAVID website (https://david-d.ncifcrf.gov/). The accession number for the RNA-seq data in this study is PRJNA961976.

### Electrophysiology analysis

The electrophysiology analysis of cardiomyocytes was performed by HELP Stem Cell Innovations Ltd. Company. The method was following the previous study 10. Briefly, cardiomyocytes were suspended and re-plated on matrigel-coated slides in 24-well plate, and continued to culture for 2 days. The current signals were recorded by EPC 10 USB—Heka Patch Clamp Amplifiers. The data was acquired using PatchMaster software, and analyzed by Clampfit software. The MEA assay was performed following the previous study 10.

### Cloning and generation of shRYR2 in hESCs lines

The generation of stable cell lines expressing shRYR2 was conducted according to the method described in a previous study 17. The psi-LVRU6P plasmid was employed for the generation of shRNA-expressing constructs. Three target sites of shRYR2 are CCGATCGTGTTGACAAAGACA, GCCTTCTATTGAGAAACGATT or CCTTATTTGTCGCATTTGCTA. The lentivirus particles were generated in 293FT cells through transfection with psi-LVRU6P-shRNA-containing plasmids, pMD2.G and psPAX2. H1 cells were transduced with the lentivirus for 48 hours, and stable shRYR2 cell lines were selected by puromycin.

### Statistical analysis

The data were presented as mean ± standard deviation (SD). A Student's t test was used for experiments with two groups; ANOVA followed by Tukey post hoc test was used for experiments with multiple groups. A p value of *p <0.05 was considered statistically significant.

## Results

### Dynamical changes of ion channels during cardiac derivation from hESCs

**Figure 1:**
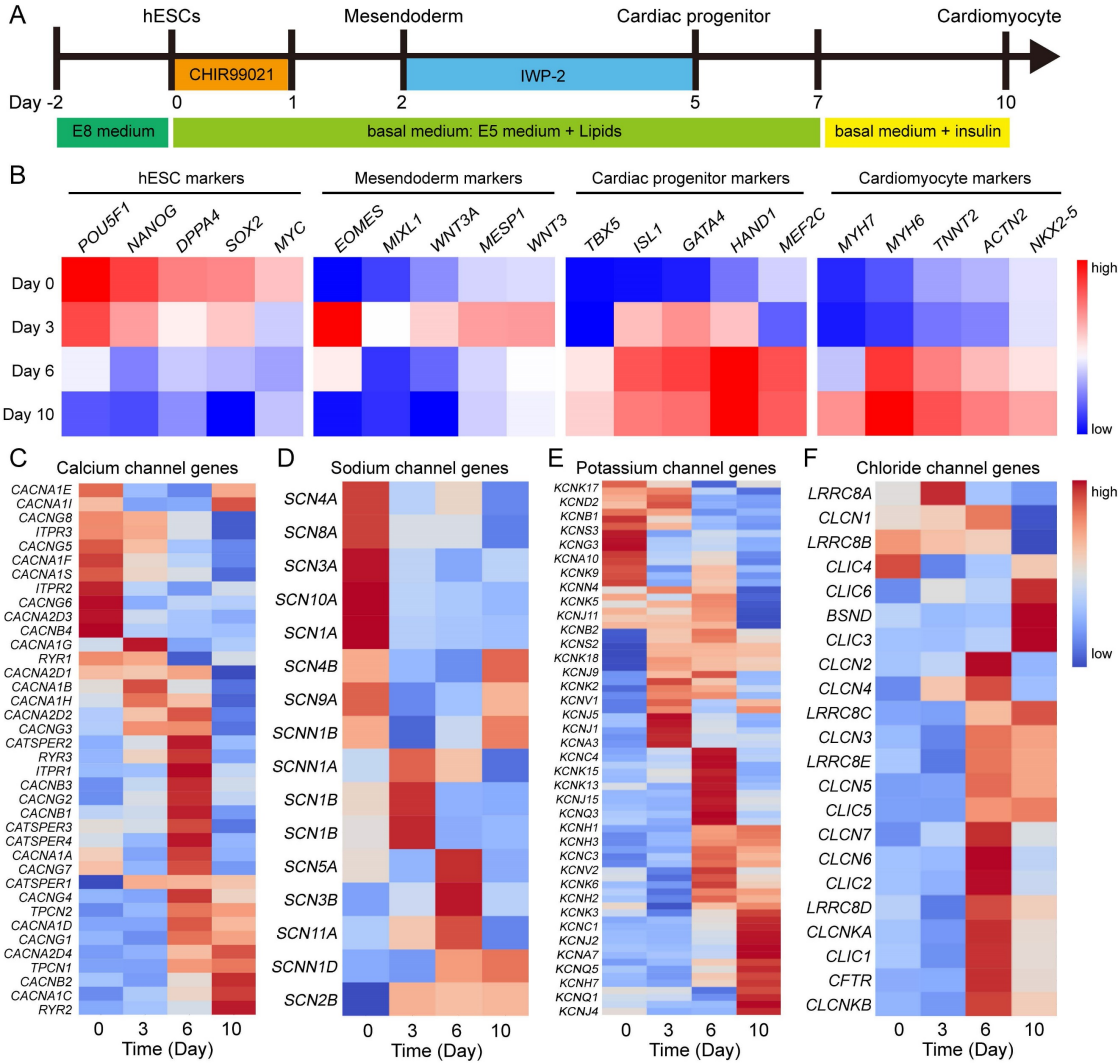
Gene expressions of ion channels show dynamical changes during cardiac differentiation. **A** Diagram of differentiation strategies for cardiac differentiation. **B** Gene expressions of hESCs, mesoendoderm, cardiac progenitor, and cardiomyocyte markers at day 0, day 3, day 6, and day 10 of differentiation. **C-F** Expression of genes encoded calcium channels (**C**), sodium channels (**D**), potassium channels (**E**), and chloride channels (**F**) in the process of cardiac differentiation.

To investigate the role of ion channels in human heart development, we utilized hESC cardiac differentiation as a model for studying embryonic heart development in human. Cardiomyocytes were generated from hESCs under chemically defined conditions (Fig. 1A). Firstly, we analyzed the microarray data of cardiac differentiation 11 to identify markers specific to different cell types. The pluripotency markers exhibited high expression at day 0 of differentiation, followed by upregulation of mesoendoderm genes at day 3 (Fig. 1B). After 6 days of differentiation, marker genes associated with cardiac progenitors and cardiomyocytes showed increased expression levels, indicating successful induction of hESCs into the cardiac lineage (Fig. 1B). These findings highlight distinct gene expression patterns during various stages of cardiac development. Furthermore, we investigated the expression patterns of calcium channels, sodium channels, potassium channels and chloride channels at different stages. Intriguingly, ion channel genes also displayed dynamic regulation during cardiac differentiation (Fig. 1C-F). Among calcium channel genes, *RYR2*, *CACNA1C*, *CACNB2*, and *CACNG1* were up-regulated, while *CACNA1S*, *CACNG5*, *ITPR3*, and *CACNG8* were down-regulated during this process (Fig. 1C). Several potassium channel genes, such as *KCNJ2*, *KCNJ4*, and *KCNQ1* displayed high expression levels in cardiomyocyte (Fig. 1E). To compare the expression of ion channels between human embryonic hearts and hESC-derived cardiomyocytes, we conducted a comprehensive analysis on the expression of ion channels at different Carnegie stages of human embryonic hearts 18 (Fig. S1). The results revealed that calcium channels, specifically *CACNA1C*, *CACNB2*, and *RYR2*, exhibited upregulation during the development of human hearts, which was consistent with hESC-derived cardiomyocyte differentiation. Similarly, other ion channels such as *SCN2B*, *SCN4A*, *SCN8A*, *KCND2*, *KCNJ2*, *KCNQ1*, *KCNJ4*, *KCNK6*, *CLIC6*, *CLIC3*, and *CLIC5* demonstrated the same expression pattern in both human embryonic heart development and hESC-derived cardiomyocytes differentiation, suggesting hESC-derived cardiomyocytes mimic the human embryonic heart development. These results demonstrated dynamic regulation of gene expressions related to ion channels during cardiac differentiation, thereby suggesting their potential involvement in determining cellular fate.

### Activation of calcium channel RyR2 induced cardiac differentiation of hPSCs

**Figure 2:**
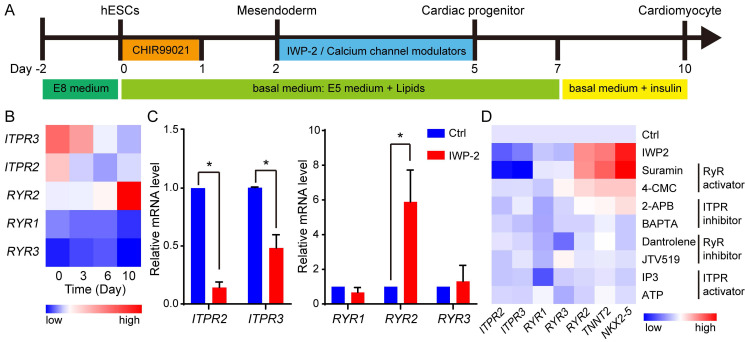
Calcium channel modulators regulated cardiomyocyte derivation from hESCs. **A** The schematic diagram showed the strategies for cardiomyocyte derivation. hESCs were treated with CHIR99021 for 1 day, and after one day, the cells were subjected to IWP-2 or calcium channel modulators from day 2 to day 5. **B** Gene expressions of *ITPR2*,* ITPR3*, *RYR1*, *RYR2*, and *RYR3* during cardiomyocyte derivation. **C** Gene expressions of *ITPR2*,* ITPR3*, *RYR1*, *RYR2*, and *RYR3* in control (Ctrl) and IWP-2 group were compared by Q-PCR at day 10 of cardiac differentiation. Blue, Ctrl; Red, IWP-2 group. Data shown are mean ± SD of three independent experiments (*p < 0.05 compared with Ctrl). **D** Calcium channel regulators were added into basal medium from day 2 to day 5 of differentiation. The expressions of the indicated genes were measured by Q-PCR, and normalized by *GAPDH*. 4-CMC, 4-Chloro-m-cresol; 2-APB, 2-aminoethoxydiphenyl borate; IP3, Inositol 1,4,5-triphosphate.

To elucidate the roles of calcium channels in cardiac differentiation of hESCs, cardiomyocytes were induced from human H1 hESCs under chemically defined conditions (Fig. 2A). The expressions of ryanodine receptors (*RYRs*) and inositol 1,4,5-triphosphate (InsP3) receptors (*ITPRs*) were analyzed throughout the process of cardiomyocyte derivation (Fig. 2B). During cardiac differentiation, *RYR2* was up-regulated while *ITPR2* and *ITPR3* were down-regulated. To validate the specific expression of RYR2 in cardiomyocytes, a comparative analysis was conducted between control cells and cardiomyocytes. The results demonstrated significantly higher expression levels of *RYR2* in cardiomyocytes; however, *ITPR2* and *ITPR3* exhibited higher expression levels in control cells (Fig. 2C). Furthermore, at the stage of WNT inhibition, a set of calcium channel regulators were subjected, and two RyR activators suramin and 4-Chloro-3-methylphenol (4-CMC) increased the expression of cardiac marker genes *NKX2-5*, *TNNT2*, and *RYR2* in the absence of WNT inhibitor (Fig. 2D). Additionally, gene expression levels of *RYR2* and *NKX2-5* were evaluated during the process of IWP- or suramin-induced cardiac differentiation. The results revealed a significant increase in their expression levels from day 3 upon treatment with either IWP-2 or suramin, while suramin exhibited an even greater upregulation of *RYR2* and *NKX2-5* compared to IWP-2 (Fig. S1E-F). These findings suggested *RYR2* may play a crucial role in cardiomyocyte generation.

**Figure 3:**
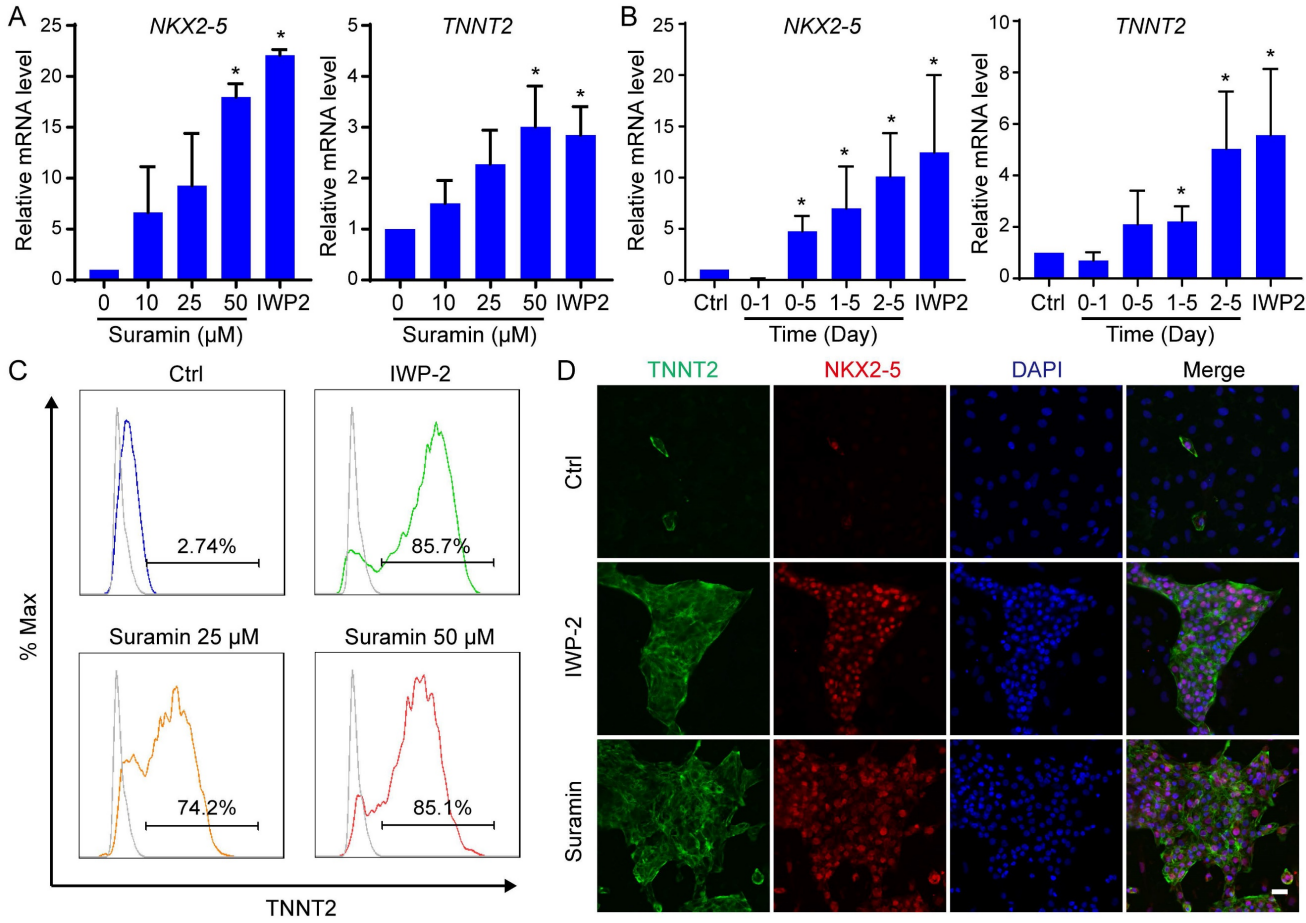
Suramin promoted cardiomyocyte derivation from hESCs. **A** Suramin exhibits a dose-dependent effect on the gene expression of *NKX2-5* and *TNNT2* during cardiac differentiation. Data shown are mean ± SD of three independent experiments (*p < 0.05 compared with suramin 0). **B** Timing effect of suramin on the gene expression of *NKX2-5* and *TNNT2* during cardiac differentiation. Suramin at a concentration of 50 μM was added at the indicated time of differentiation. Data shown are mean ± SD of three independent experiments (*p < 0.05 compared with none-treated control). **C** Flow cytometry analysis showed the TNNT2-positive population after 12 days of differentiation. IWP-2 3 μM, suramin 25 μM, or suramin 50 μM was subjected to basal medium from day 2 to day 5 of differentiation. **D** Immunostaining images showed the levels of TNNT2 (green), NKX2-5 (red) and DAPI (blue) in the differentiated cells. IWP-2 (3 μM) or suramin (50 μM) was added to basal medium from day 2 to day 5 of differentiation. Scale bar, 50 μm.

We further validated the impact of RYR2 activator, suramin, on determining the fate of cardiomyocyte. The effect of suramin on cardiac induction in hESCs was dose- and time-dependent (Fig. 3A-B). An optimal concentration of 50 μM suramin resulted in a significantly elevated expression level of cardiac markers (Fig. 3A). A previous study has reported that adding suramin with CHIR99021 on day 0-2 suppressed cardiac differentiation 19. Consistent with this finding, we also observed a reduction in cardiomyocyte markers when suramin was administered during the initial stage (day 0-1) (Fig. 3B). Therefore, the appropriate timing for suramin treatment started from day 2 of differentiation and continued for 3-5 days (Fig. 3B). Remarkably, when exposed to a concentration of 50 μM from day 2 to day 5, more than 80% of hESCs successfully differentiated into functional cardiomyocytes (Fig. 3C). The efficient differentiation induced by suramin was confirmed through immunostaining targeting TNNT2 (Fig.3D and S2E). Furthermore, we successfully employed these optimized procedures using suramin to induce cardiomyocyte differentiation in H9 hESC and NL4 iPSC lines (Fig. S2A-D). Together, the data indicated that suramin efficiently induced cardiac differentiation in the absence of WNT inhibitor.

### Modulation of the chloride channels induced cardiac differentiation of hPSCs

**Figure 4:**
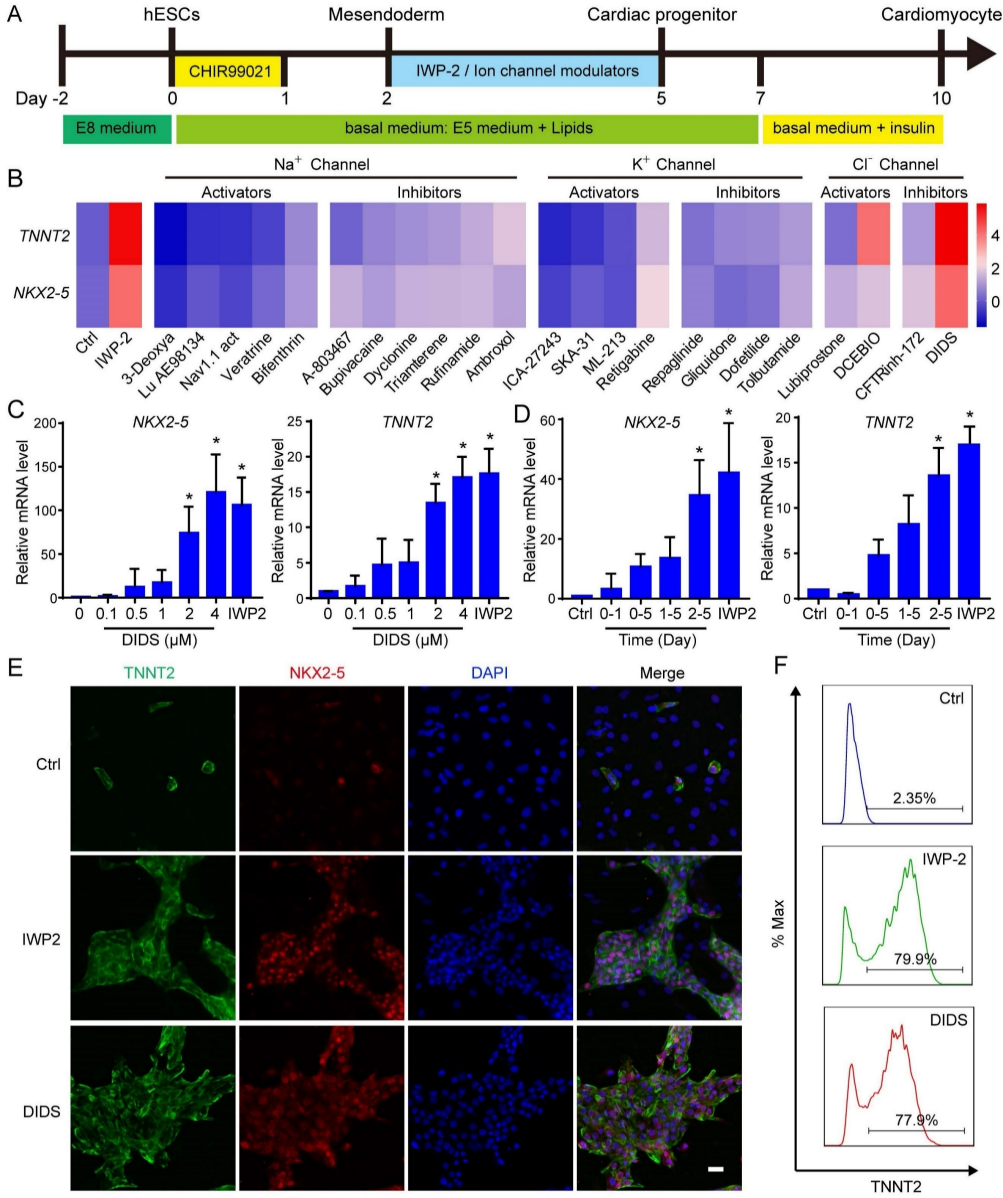
Chloride channel modulator DIDS induced cardiomyocytes from hESCs. **A** The schematic diagram of cardiomyocyte derivation. The cells were subjected to IWP-2 or the indicated ion channel modulators from day 2 to day 5. **B** Effect of Sodium, potassium, and chloride channels on cardiac differentiation. The indicated ion channel modulators were applied from day 2 to day 5 of cardiac differentiation. Gene expressions of *TNNT2* and *NKX2-5* were determined by Q-PCR at day 10. The scale bar indicated the log2 fold change compared with Ctrl. The doses of ion channel modulators were listed in supplemental table. **C** Dose-dependent effect of DIDS on the gene expression of *NKX2-5* and *TNNT2* during cardiomyocyte derivation. Data shown are mean ± SD of three independent experiments (*p < 0.05 compared with DIDS 0). **D** Timing effect of DIDS on the gene expression of *NKX2-5* and *TNNT2* during cardiac differentiation. DIDS at a concentration of 4 μM was added at the indicated time of differentiation. Data shown are mean ± SD of three independent experiments (*p < 0.05 compared with Ctrl). **E** Immunostaining images showed the levels of TNNT2 (green), NKX2-5 (red) and DAPI (blue) in the differentiated cells after 12 days of differentiation. IWP-2 (3 μM) or DIDS (4 μM) was added to basal medium from day 2 to day 5 of differentiation. Scale bar, 50 μm. **F** Flow cytometry analysis showed the TNNT2-positive population after 12 days of differentiation. IWP-2 (3 μM) or DIDS (4 μM) was added to basal medium from day 2 to day 5 of differentiation.

After elucidating the role of RYR2 calcium channel in cardiac differentiation, we proceeded to investigate the potential involvement of other ion channels in determining cardiac cell fate. Various modulators targeting sodium channels, potassium channels or chloride channels were applied to mesodermal cells from day 2 to day 5 of differentiation (Fig. 4A). Among these ion channel modulators, only DIDS, a chloride channel inhibitor, significantly enhanced cardiac differentiation as evidenced by upregulated expression of *NKX2-5* and *TNNT2* (Fig. 4B and S3). Furthermore, we demonstrated that the induction of cardiomyocytes by DIDS was dose-dependent with an optimal concentration at 4 μM (Fig. 4C). The effect of DIDS also exhibited time-dependency, and the best induction was achieved between day 2 and day 5 (Fig. 4D). The efficacy of cardiac differentiation was confirmed through flow cytometry analysis and immunostaining for NKX2-5 and TNNT2 (Fig. 4E-F). Successful generation of cardiomyocytes from H9 hESCs and NL4 iPSCs was also achieved using DIDS treatment (Fig. S4A-D).

A previous study has reported that 1-ethyl-2-benzimidazolinone (EBIO), an activator of Ca2^+^ sensitive K^+^ channels, enhanced the differentiation of cardiomyocytes from hPSCs by inducing cell loss and promoting cardiac progenitor preservation 20. To investigate the impact of suramin and DIDS on cell survival, we monitored the cell number during hESC differentiation (Fig. S4E-F). The results revealed no significant differences in cell survival had among the control, IWP-2, suramin, and DIDS groups (Fig. S4E-F). Additional cardiac markers were determined to confirm the induction of cardiomyocytes from hESCs by suramin, or DIDS (Fig. S4G). Interestingly, compared with IWP2, both suramin and DIDS significantly increased expression level of *NPPA*, an atrial-like cardiomyocyte marker; moreover, suramin up-regulated *HCN4* gene expression as well, a nodal-like marker (Fig. S4G). These findings suggested that suramin and DIDS may induce different subtypes of cardiomyocytes. Furthermore, after 40 days of differentiation, we observed normal sarcomere structures in the cardiomyocytes induced by either suramin or DIDS (Fig. 4H). These results confirmed that both suramin and DIDS promoted the differentiation of hESCs into cardiomyocytes.

### Suramin and DIDS induced cardiomyocyte differentiation from hPSC by inhibiting WNT pathway and activating RYR2

**Figure 5:**
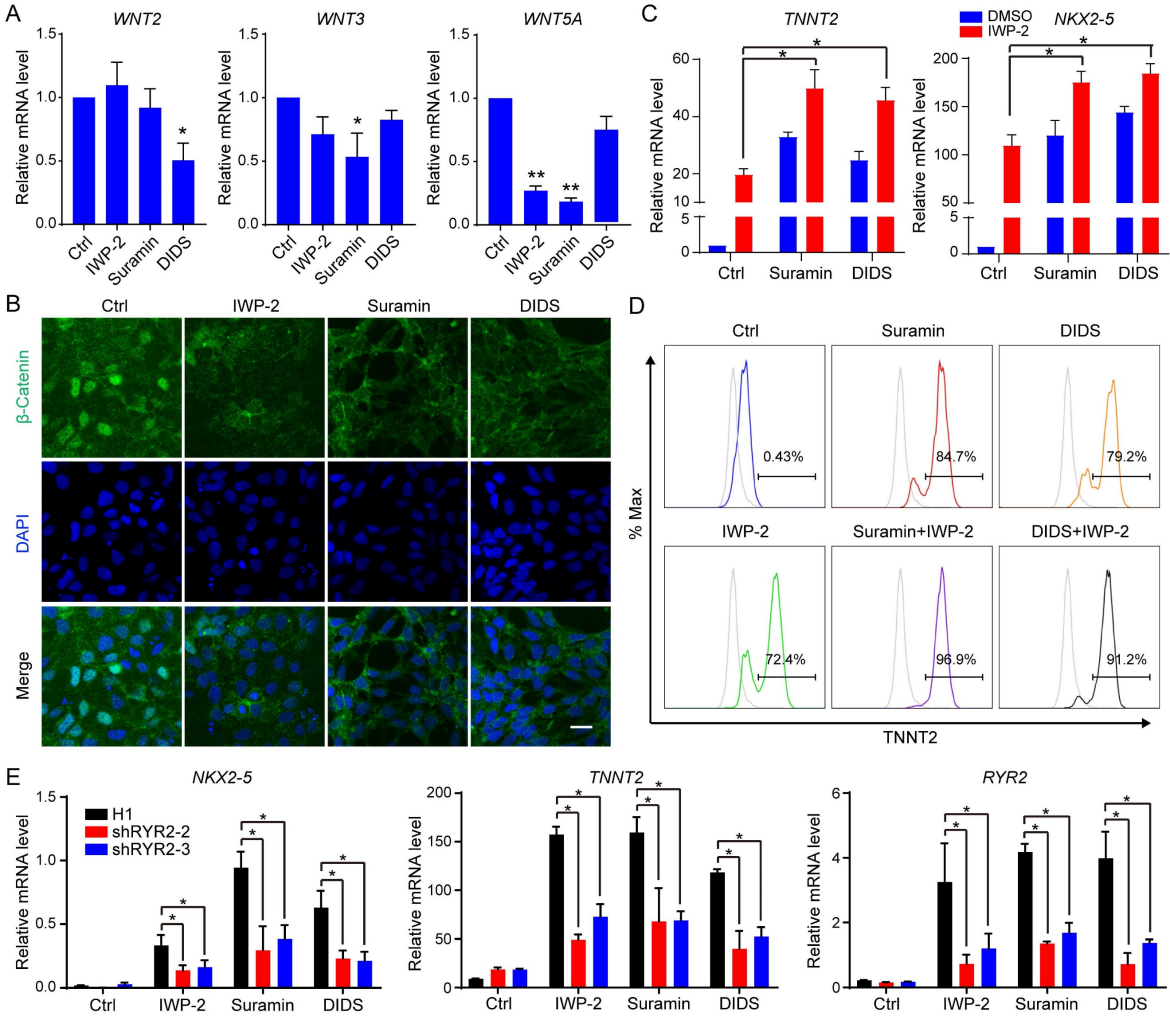
The induction of cardiac differentiation by Suramin and DIDS was partially reliant on WNT inhibition and RYR2. **A** Effect of Suramin and DIDS on *WNTs* expression. hESCs were treated with CHIR99021 for 1 day, and then subjected to IWP-2 (3 μM), suramin (50 μM), or DIDS (4 μM) for 24 hours. Gene expression of *WNTs* were analyzed by Q-PCR. Data shown are mean ± SD of three independent experiments (*p < 0.05, **p < 0.01 compared with Ctrl). **B** Immunostaining images of β-Catenin (Green) and DAPI (Blue) in the indicated groups. Scale bar, 20 μm. **C** Suramin or DIDS promoted IWP-2 induced cardiac differentiation. hESCs were treated with IWP-2, suramin, DIDS, suramin+IWP-2, DIDS+IWP-2 at day 2 to day 5 of differentiation. Gene expression of *TNNT2* and *NKX2-5* were analyzed by Q-PCR. Data shown are mean ± SD of three independent experiments (*p < 0.05 compared with IWP-2). **D** Flow cytometry analysis showed the TNNT2-positive population after 12 days of differentiation in the indicated groups. **E** RYR2 knockdown resulted in decreased gene expression of *NKX2-5*, *TNNT2* and *RYR2* in cardiomyocytes induced by IWP-2 (3 μM), suramin (50 μM), or DIDS (4 μM) on day 10 of differentiation. Data shown are mean ± SD of three independent experiments (*p < 0.05 compared to the indicated group).

Suramin is a versatile chemical compound with multiple potential targets, including Sirtuins, JNK, ERK, and P2Y receptor 21-23. To elucidate the mechanism underlying suramin-induced cardiac differentiation, we used the inhibitors targeting these signaling pathways to assess their impact on cardiomyocyte differentiation from hESCs; however, none of them induced the expression of cardiac markers (Fig. S5A-B). This data suggested that suramin-triggered cardiac differentiation was not dependent on its inhibition of Sirtuins, JNK, ERK and P2Y receptor. WNT pathway plays a critical role in cardiac cell fate determination 5, 6. Previous studies reported that both suramin and DIDS regulated WNT signaling pathway 24-26. Therefore, we explored the influence of suramin or DIDS on WNT pathway during cardiac differentiation. Our results showed that suramin downregulated the expression of *WNT5A* and *WNT3*, while DIDS decreased *WNT2* expression (Fig. 5A). Immunostaining analysis revealed that both suramin and DIDS effectively inhibited the nuclear translocation of β-catenin, similar to IWP-2 (Fig. 5B), suggesting that suramin and DIDS partially inhibited WNT pathway in cardiac cell fate determination. Furthermore, we evaluated the combined effect of IWP-2 with either suramin or DIDS on cardiac differentiation, and found that both compounds significantly increased gene expression of cardiac markers along with IWP-2 (Fig. 5C-D), indicating involvement of additional signaling pathways in cardiomyocyte generation.

Both suramin and DIDS have been show to activate RYR channel, and elevate intracellular calcium level 27, 28. To confirm their effect on the RYR2 channel, we monitored intracellular calcium concentration using the calcium fluorescent indicator Calbryte™ 590 AM. Following treatment with suramin or DIDS, a significant increase in intracellular calcium was observed (Fig. S5C). Subsequently, we employed the calcium chelator BAPTA to downregulate calcium concentration during suramin or DIDS treatment, and observed that alterations in calcium level slightly impeded cardiac differentiation (Fig. S5E-F), suggesting a partial dependence of suramin and DIDS on modulating intracellular calcium for their effect on cardiac differentiation. To further clarify the impact of RYR2 on cardiac differentiation, stable cell lines with knockdown of RYR2 were established (Fig. S5F-H). Our results demonstrated that knockdown of RYR2 significantly attenuated the expression of cardiac markers in IWP-2, DIDS, or Suramin-induced cardiomyocytes (Fig. 5E), highlighting the pivotal role of RYR2 as a key regulator in cardiac differentiation.

### Verification of cardiomyocytes induced by ion channel modulation

**Figure 6:**
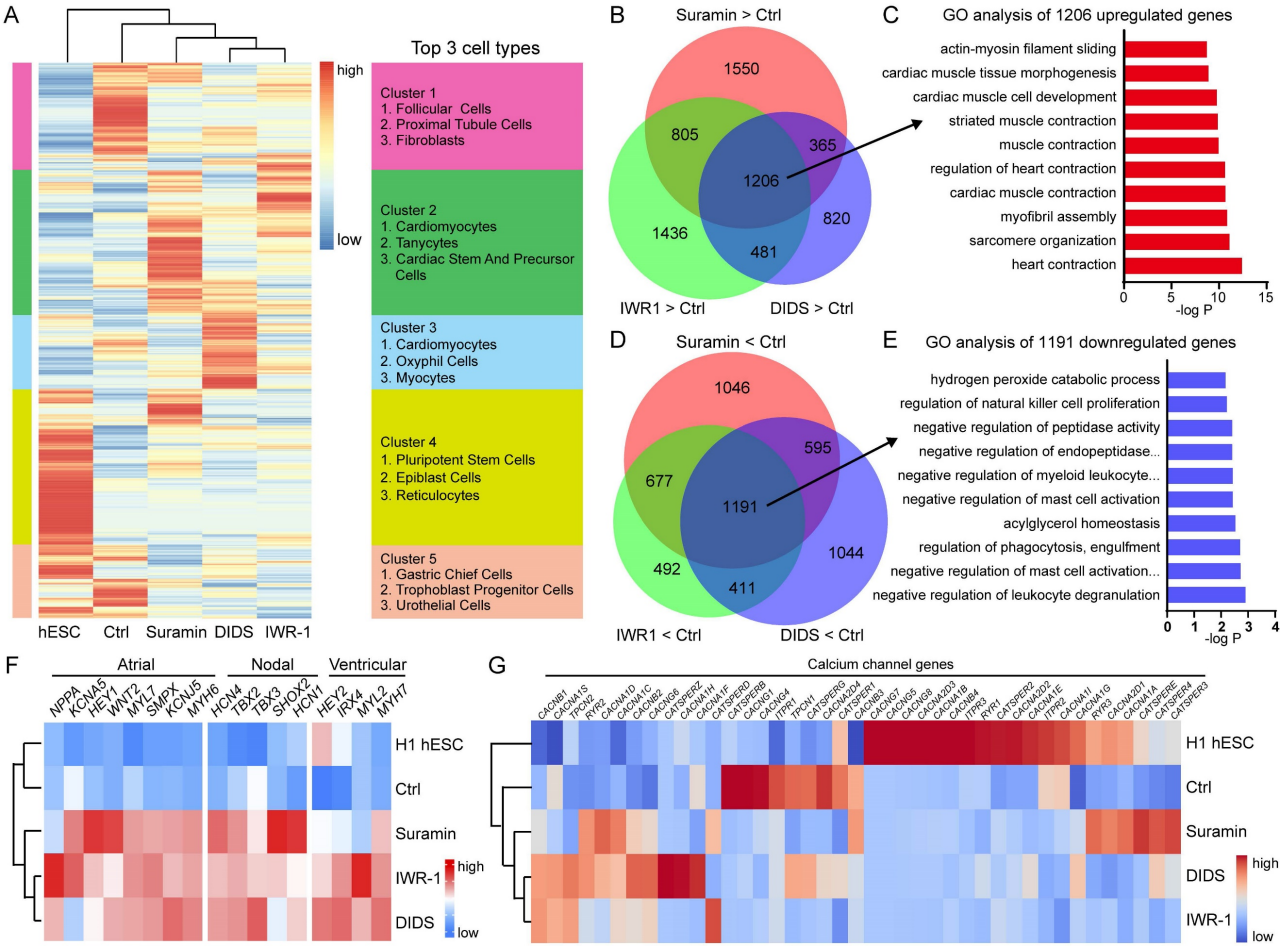
Analysis of gene expression of cardiomyocytes induced by ion channel modulators. **A** Cluster analysis of RNA-sequencing data. IWR-1 (5 μM), DIDS (4 μM) or suramin (50 μM) was added to basal medium for 3 days. Samples were harvested for RNA-sequencing after 10 days of differentiation. **B** Venn diagram showing the differentially expressed genes (DEGs) up-regulated by suramin, DIDS or IWR-1. **C** Gene ontology analysis of the overlapped 1206 genes up-regulated by suramin, DIDS, or IWR-1 group. **D** Comparison of the down-regulated DEGs by suramin, DIDS, or IWR-1 group VS non-treated control. **E** Gene ontology analysis of the overlapped 1191 genes down-regulated by suramin, DIDS, or IWR-1 group. **F-G** Heatmaps showing the expressions of genes related to different cardiomyocyte subtypes (**F**) and calcium channels (**G**).

Gene expression profiles obtained from RNA-seq were compared among hESCs, control, IWR1, suramin, and DIDS-induced cardiomyocytes. Hierarchical clustering analysis showed that the gene expression profiles of IWR1, DIDS, or suramin-treated samples exhibited closer similarity to each other than to those of control or hESCs (Fig. 6A). Additionally, Enrichr analysis was performed on the clustered genes 29, which showed that treatment with suramin, DIDS, or IWR-1 resulted in increased expression of genes enriched in cardiomyocytes and cardiac precursor cells (Fig. 6A). The Venn diagram demonstrated that there were 1206 overlapping up-regulated genes in the suramin, DIDS, and IWR-1 group (Fig. 6B), which were associated with heart structure and development (Fig. 6C).

Furthermore, a total of 1191 immune cell-enriched genes were down-regulated in the suramin, DIDS, and IWR-1 group compared to the non-treated control (Fig. 6D-E). Subsequently, KEGG pathway analyses demonstrated that pathways related to heart functions were up-regulated in all three conditions (Fig. S6A-C). Notably, the calcium signaling pathway was only observed in both suramin and DIDS-treated groups (Fig. S6A-B), indicating a critical role for calcium signaling in cardiomyocyte induction by ion channel modulator. The gene expression pattern of heart-related genes further supported the point that ion channel modulations by suramin and DIDS promoted the derivation of cardiomyocytes (Fig. 6F). Moreover, we also found that suramin-induced cardiomyocytes expressed high level of nodal markers such as *HCN4*, *SHOX2*, and *TBX18*. (Fig. 6F). In addition, the expression of ion channels was also analyzed, which revealed that IWR1 and DIDS-induced cardiomyocytes had similar expression of calcium channels, but suramin-induced cardiomyocytes had a unique gene expression of calcium channels (Fig. 6G). The gene expression of chloride channels showed a similar pattern as calcium channels, but not sodium and potassium channels (Fig. S6D-F). Furthermore, we conducted an analysis of the down-regulated genes in the suramin, DIDS, or IWR-1 groups compared to the control group. The results revealed that these down-regulated genes were associated with lipid metabolism and blood vessel development, and similar down-regulated pathways were observed across all three groups (Fig. S6G). Collectively, the global gene expression profiles proved that suramin or DIDS induced cardiomyocytes from hPSCs.

### Calcium or chloride channel modulators induced functional cardiomyocyte from hESCs

**Figure 7:**
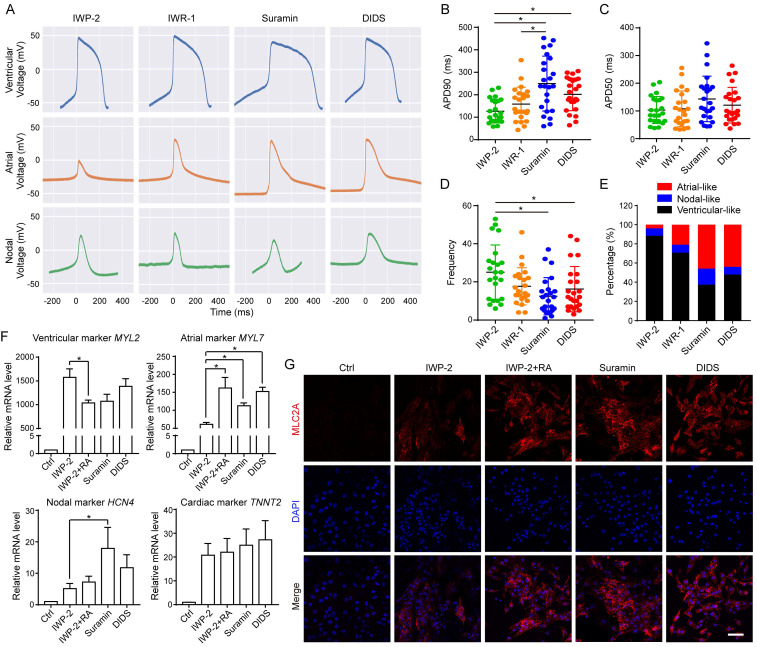
Electrophysiological analysis of suramin, DIDS or WNT inhibitors-induced cardiomyocytes. **A** The action potential of ventricular-, atrial- and nodal-like cardiomyocytes in the indicated groups. IWP-2 (3 μM), IWR-1 (5 μM), suramin (50 μM), or DIDS (4 μM) was added to basal medium for 3 days. The action potential was recorded at day 40-50. **B-D** Quantification of action potential duration (APD)90, APD50, Frequency from the electrophysiology analysis. N=24-25 for each group. Data shown are mean ± SD, *p < 0.05 compared with the indicated group. **E** Percentage of ventricular-like, nodal-like, and atrial-like action potentials were quantified in the cardiomyocytes induced by IWP-2, IWR-1, suramin, or DIDS. N=24-25 for each group. **F** Gene expressions of cardiomyocyte subtype markers were determined by Q-PCR at day 40-50 of differentiation. Data shown are mean ± SD of three independent experiments (*p < 0.05 compared with IWP-2 group). **G** Immunostaining images represented the level of MLC2A (red) and DAPI (blue) in the differentiated cells at day 40-50 of differentiation. Scale bar, 50 μm.

hESCs can differentiate into several types of functional cardiomyocytes such as atrial-like, ventricular-like, and nodal-like cardiomyocytes. To understand the electrophysiological function of calcium or chloride channel modulators induced cardiomyocytes, we analyzed the action potential (AP) of cardiomyocytes derived using WNT inhibitors or ion channel modulators. Suramin and DIDS groups showed typical electrophysiological signals of atrial-like, ventricular-like, and nodal-like cardiomyocytes (Fig. 7A). Suramin-induced cardiomyocytes had longer action potential duration (APD) 90 than WNT inhibitor groups (Fig. 7B), but had no effect on APD50 (Fig. 7C). Cardiomyocytes of suramin or DIDS group showed shorter frequency compared with those of IWP-2 group (Fig. 7D). Based on the electrophysiological signals, we defined the ventricular-like (APD90/50 < 1.4), Nodal like (1.7 < APD90/50 < 1.4), and atrial-like (APD90/50 > 1.7) cardiomyocytes 30, 31, and the data indicated that suramin and DIDS groups had more atrial-like cardiomyocytes than WNT inhibitor groups (Fig. 7E). Multielectrode array (MEA) assay revealed the fluctuations in extracellular field potential generated from DIDS or suramin-induced cardiomyocytes (Fig. S7A-B).

To provide a comprehensive evaluation of cardiomyocyte subtypes, we analyzed the gene expressions of ventricular-like cardiomyocyte markers (*IRX4* and *MYL2*), atrial -like cardiomyocyte markers (*NPPA* and *MYL7*), nodal-like cardiomyocyte marker (*HCN4*) and common cardiomyocyte marker (*TNNT2*) at approximately 40-50 days of differentiation (Fig. 7F, S7C-D). Our results demonstrated that the expressions of atrial cardiomyocyte markers, *MYL7* and *NPPA*, were upregulated by suramin and DIDS, similar to the effect observed with IWP-2 plus RA (Fig. 7F and S7D). Additionally, suramin induced high level of nodal-like cardiomyocyte marker *HCN4* (Fig. 7F). Immunostaining for atrial-like cardiomyocyte marker MLC2A encoded by *MYL7*gene showed that suramin and DIDS induced more MLC2A positive cells than IWP-2 (Fig. 7G). Additionally, immunostaining for nodal-like cardiomyocyte marker HCN4 revealed that suramin treatment led to an elevated level of HCN4 expression compared to IWP-2 treatment (Fig. S7E), which was consistent with the qPCR result. Collectively, these results suggested that ion channel modulators have the potential to induce functional cardiomyocytes, and generate a higher proportion of non-ventricular-like cells when compared to the classical WNT inhibitors, thus providing unique strategies for driving cardiac induction in hESCs.

## Discussion

Ion channels play crucial roles in maintaining the functionality of cardiomyocytes, encompassing sodium, potassium, calcium and chloride channels that are expressed in cardiomyocytes 32-35. Throughout human and mouse cardiac development, the expression of ion channels undergoes dynamic regulation; however, their precise contribution to cardiomyocyte generation remains elusive. hESCs offer a valuable platform for investigating the impact of ion channels on cardiomyocyte derivation during human embryogenesis. Our study demonstrated that the regulation of calcium and chloride channels was implicated in the generation of human cardiomyocytes from hESCs, indicating the significant roles played by ion channels in human embryonic heart development.

During heart development, there are alterations in the profiles of ion channels in heart tissues. In mouse heart, calcium channels (*Cacna1c*, *Cacnb2*, *Cacna2d1*, *Plb*, *Ryr2*, and *Serca2*) were upregulated during perinatal maturation 36. Similarly, dynamic regulation of ion channels has been observed across different Carnegie stages of human hearts 18 (Fig. S1A). In our study, the expression levels of CACNB2, CACNA1C, and RYR2 were high in the process of cardiomyocyte generation from hESCs (Fig. 1C and S1A), which was consistent in mouse and human hearts. Besides calcium channels, sodium, potassium and chloride channels also exhibited patterned expression. Sodium channel genes *Scn5a* and *Scn7a* were highly expressed in adult mouse hearts 36 and late stage of human embryonic heart 18, which was not detected in hESC-derived cardiomyocytes (Fig. 1D and S1D). The CLC family of chloride channel transcripts (*Clic1* and *Clic3*) as well as potassium channel transcripts (*Kcnj2*, *Kcnh2*, and *Kcnk5*) demonstrated comparable expression patterns across mouse 36, human hearts 18, and hESC-derived cardiomyocytes (Fig. 1E and S1C). Further investigations are worth to elucidate the precise roles of ion channels during embryonic heart development.

To investigate the roles of ion channels in human cardiomyocyte generation, we employed various ion channel modulators and discovered that suramin and DIDS effectively induced the differentiation of hESC into cardiomyocytes partially through RYR2 (Fig. 5 and S5). Knockout of RYR2 in hiPSCs diminished the viability and contractile function of differentiated cardiomyocytes 37, implying a pivotal role for RYR2 in human embryonic heart development. The impact of RYR2 on mouse cardiac development has been investigated through whole-body or cardiac-specific knockout of RYR2 *in vivo*. Whole-body *Ryr2* knockout mice die on embryonic day 10 with abnormal heart tube formation 13, and heart-specific *Ryr2* knockout mice exhibited functional and structural hallmarks of heart failure 38. Employing Cas9/AAV9-mediated *RYR2* knockout in cardiomyocytes resulted in impaired cardiomyocyte maturation characterized by ultrastructural and transcriptomic defects, which were associated with activation of ER stress pathways 39. Together, these findings highlight the crucial roles played by RYR2 in both human and mouse heart development.

Besides RYR2, other ion channels are implicated in the process of cardiomyocyte lineage commitment. Inhibition of the L-type calcium channel by nifedipine suppressed cardiac lineage commitment in mESCs 14. Additionally, activation of small-/intermediate-conductance Ca^2+^-activated potassium channels (SKs) by 1-ethyl-2-benzimidazolinone (EBIO) drove mESCs towards cardiomyocytes 15. EBIO also promotes the differentiation of cardiomyocytes from hPSCs by inducing cell loss and promoting cardiac progenitor preservation 20. In contrast to EBIO, ion channel regulators such as suramin or DIDS enhance cardiac differentiation without inducing cell loss (Fig. S4E-F). Deletion of the sodium-calcium exchanger (*Ncx1*) in mice resulted in absence of heartbeat and impaired embryo development 12. Furthermore, inhibition of NCX1 using CB-DMB suppressed mESC differentiation into cardiomyocytes 40. We also investigated various sodium, potassium, calcium, and chloride ion channel regulators for their roles in cardiac differentiation; however, none except suramin and DIDS effectively induced cardiomyocytes (Fig. 4 and S3). Given the unique expression pattern of ion channels in cardiomyocytes, further comprehensive investigations are worth to elucidate the intrinsic relationship between specific ion channels and human cardiomyocyte development.

Suramin, a multi-target drug, exerts inhibitory effects on ERK, SIRT, TGFβ, JNK and P2Y2 signaling pathways while activating RYR channels 21-23. To investigate its impact on cardiac differentiation, we employed inhibitors targeting these pathways; however, none of them induced the expression of cardiac markers (Fig. S5A-B). Additionally, suramin was found to inhibit WNT signaling 24, which plays a critical role in cardiac differentiation. During the early stage of differentiation, activation of GSK3β/WNT pathway by CHIR99021 promoted hESC differentiate into mesoderm; subsequently, WNT inhibitors facilitated the generation of cardiomyocyte progenitors 5, 6. Following induction of mesoderm, suramin and DIDS decreased gene expression levels of WNTs and suppressed nuclear localization of β-catenin (Fig. 5A-B), indicating inhibition of WNT pathway. A previous study demonstrated that adding suramin with CHIR99021 from day 0-2 inhibited cardiac differentiation 19. In our study, initiating suramin treatment from day 0 didn't achieve optimal differentiation efficiency; instead, the appropriate timing for suramin treatment started from day 2 and continued for 3-5 days during differentiation (Fig. 3B), consistent with previous findings. However, the effect of suramin and DIDS on cardiac differentiation partially extended beyond the regulation of the WNT pathway, as their ability to induce cardiomyocyte generation from hESCs was impaired upon knockdown of *RYR2* (Fig. 5E and S5C-E). These data suggested that suramin and DIDS regulated cardiomyocyte generation through both WNT inhibition and RYR2 activation.

Suramin is a versatile molecule with diverse potential applications, encompassing parasitic and viral diseases, autism, and cancer 21. Additionally, the suramin analogs NF340 and NF546 exhibited cardioprotective effects in a murine model of heart graft rejection 41. Our study unveiled that suramin promotes human embryonic cardiomyogenesis by inhibiting WNT signaling while activating RYR2 channels. Collectively, these findings suggested the potential application of suramin in treating cardiovascular diseases.

In summary, we have elucidated the expression patterns of ion channels during cardiomyogenesis from hPSCs, and investigated the impact of ion channel modulators on cardiac differentiation. Our study has demonstrated that ion channels play a pivotal role in regulating human cardiomyocyte development and proposes novel strategies for inducing cardiomyocytes from hPSCs through modulation of calcium or chloride channels. This innovative approach holds great promise as a potential alternative or adjunctive method to enhance cardiomyocyte derivation in regenerative medicine.

## Supplementary Material

Supplementary figures and tables.

## Abbreviations

hESCs: human embryonic stem cells
hPSCs: human pluripotent stem cells
RYR2: ryanodine receptor 2
CHD: congenital heart defects
mESCs: mouse embryonic stem cells
EBIO: 1-ethyl-2-benzimidazolinone
ITPRs: inositol 1, 4, 5-triphosphate (InsP3) receptors
4-CMC: 4-Chloro-3-methylphenol
AP: action potential
APD: action potential duration
MEA: Multielectrode array
PFA: paraformaldehyde
SD: standard deviation

## References

[1] Davies MP, An RH, Doevendans P, Kubalak S, Chien KR, Kass RS (1996). Developmental changes in ionic channel activity in the embryonic murine heart. Circ Res.

[2] Conforti L, Tohse N, Sperelakis N (1993). Tetrodotoxin-sensitive sodium current in rat fetal ventricular myocytes-contribution to the plateau phase of action potential. J Mol Cell Cardiol.

[3] Boczek NJ, Ye D, Jin F, Tester DJ, Huseby A, Bos JM (2015). Identification and Functional Characterization of a Novel CACNA1C-Mediated Cardiac Disorder Characterized by Prolonged QT Intervals With Hypertrophic Cardiomyopathy, Congenital Heart Defects, and Sudden Cardiac Death. Circ Arrhythm Electrophysiol.

[4] Izarzugaza JMG, Ellesoe SG, Doganli C, Ehlers NS, Dalgaard MD, Audain E (2020). Systems genetics analysis identifies calcium-signaling defects as novel cause of congenital heart disease. Genome Med.

[5] Naito AT, Shiojima I, Akazawa H, Hidaka K, Morisaki T, Kikuchi A (2006). Developmental stage-specific biphasic roles of Wnt/beta-catenin signaling in cardiomyogenesis and hematopoiesis. Proc Natl Acad Sci U S A.

[6] Lian X, Hsiao C, Wilson G, Zhu K, Hazeltine LB, Azarin SM (2012). Robust cardiomyocyte differentiation from human pluripotent stem cells via temporal modulation of canonical Wnt signaling. Proc Natl Acad Sci U S A.

[7] Burridge PW, Keller G, Gold JD, Wu JC (2012). Production of de novo cardiomyocytes: human pluripotent stem cell differentiation and direct reprogramming. Cell stem cell.

[8] Lian X, Zhang J, Azarin SM, Zhu K, Hazeltine LB, Bao X (2013). Directed cardiomyocyte differentiation from human pluripotent stem cells by modulating Wnt/beta-catenin signaling under fully defined conditions. Nature protocols.

[9] Menendez L, Yatskievych TA, Antin PB, Dalton S (2011). Wnt signaling and a Smad pathway blockade direct the differentiation of human pluripotent stem cells to multipotent neural crest cells. Proc Natl Acad Sci U S A.

[10] Meng Y, Song C, Ren Z, Li X, Yang X, Ai N (2021). Nicotinamide promotes cardiomyocyte derivation and survival through kinase inhibition in human pluripotent stem cells. Cell Death Dis.

[11] Lin Y, Linask KL, Mallon B, Johnson K, Klein M, Beers J (2017). Heparin Promotes Cardiac Differentiation of Human Pluripotent Stem Cells in Chemically Defined Albumin-Free Medium, Enabling Consistent Manufacture of Cardiomyocytes. Stem Cells Transl Med.

[12] Koushik SV, Wang J, Rogers R, Moskophidis D, Lambert NA, Creazzo TL (2001). Targeted inactivation of the sodium-calcium exchanger (Ncx1) results in the lack of a heartbeat and abnormal myofibrillar organization. FASEB J.

[13] Takeshima H, Komazaki S, Hirose K, Nishi M, Noda T, Iino M (1998). Embryonic lethality and abnormal cardiac myocytes in mice lacking ryanodine receptor type 2. EMBO J.

[14] Nguemo F, Fleischmann BK, Gupta MK, Saric T, Malan D, Liang H (2013). The L-type Ca2+ channels blocker nifedipine represses mesodermal fate determination in murine embryonic stem cells. PLoS One.

[15] Kleger A, Seufferlein T, Malan D, Tischendorf M, Storch A, Wolheim A (2010). Modulation of calcium-activated potassium channels induces cardiogenesis of pluripotent stem cells and enrichment of pacemaker-like cells. Circulation.

[16] Song C, Xu F, Ren Z, Zhang Y, Meng Y, Yang Y (2019). Elevated Exogenous Pyruvate Potentiates Mesodermal Differentiation through Metabolic Modulation and AMPK/mTOR Pathway in Human Embryonic Stem Cells. Stem Cell Reports.

[17] Zhang Y, Xu J, Ren Z, Meng Y, Liu W, Lu L (2021). Nicotinamide promotes pancreatic differentiation through the dual inhibition of CK1 and ROCK kinases in human embryonic stem cells. Stem Cell Res Ther.

[18] VanOudenhove J, Yankee TN, Wilderman A, Cotney J (2020). Epigenomic and Transcriptomic Dynamics During Human Heart Organogenesis. Circ Res.

[19] Fort L, Gama V, Macara IG (2022). Stem cell conversion to the cardiac lineage requires nucleotide signalling from apoptosing cells. Nat Cell Biol.

[20] Jara-Avaca M, Kempf H, Ruckert M, Robles-Diaz D, Franke A, de la Roche J (2017). EBIO Does Not Induce Cardiomyogenesis in Human Pluripotent Stem Cells but Modulates Cardiac Subtype Enrichment by Lineage-Selective Survival. Stem Cell Reports.

[21] Wiedemar N, Hauser DA, Maser P (2020). 100 Years of Suramin. Antimicrob Agents Chemother.

[22] Wang A, Wang J, Wu J, Deng X, Zou Y (2019). Suramin protects hepatocytes from LPS-induced apoptosis by regulating mitochondrial stress and inactivating the JNK-Mst1 signaling pathway. J Physiol Sci.

[23] Liu N, He S, Tolbert E, Gong R, Bayliss G, Zhuang S (2012). Suramin alleviates glomerular injury and inflammation in the remnant kidney. PLoS One.

[24] Koval A, Ahmed K, Katanaev VL (2016). Inhibition of Wnt signalling and breast tumour growth by the multi-purpose drug suramin through suppression of heterotrimeric G proteins and Wnt endocytosis. Biochem J.

[25] Zhang W, Zhang H, Wang N, Zhao C, Zhang H, Deng F (2013). Modulation of beta-catenin signaling by the inhibitors of MAP kinase, tyrosine kinase, and PI3-kinase pathways. Int J Med Sci.

[26] Yang X, Tang S, Li D, Yu X, Wang F, Xiao X (2018). DIDS inhibits overexpression BAK1-induced mitochondrial apoptosis through GSK3beta/beta-catenin signaling pathway. J Cell Physiol.

[27] Sitsapesan R (1999). Similarities in the effects of DIDS, DBDS and suramin on cardiac ryanodine receptor function. J Membr Biol.

[28] O'Neill ER, Sakowska MM, Laver DR (2003). Regulation of the calcium release channel from skeletal muscle by suramin and the disulfonated stilbene derivatives DIDS, DBDS, and DNDS. Biophys J.

[29] Chen EY, Tan CM, Kou Y, Duan Q, Wang Z, Meirelles GV (2013). Enrichr: interactive and collaborative HTML5 gene list enrichment analysis tool. BMC Bioinformatics.

[30] Burridge PW, Matsa E, Shukla P, Lin ZC, Churko JM, Ebert AD (2014). Chemically defined generation of human cardiomyocytes. Nat Methods.

[31] Churko JM, Garg P, Treutlein B, Venkatasubramanian M, Wu H, Lee J (2018). Defining human cardiac transcription factor hierarchies using integrated single-cell heterogeneity analysis. Nat Commun.

[32] Duan DD (2011). The ClC-3 chloride channels in cardiovascular disease. Acta Pharmacol Sin.

[33] Abriel H, Rougier JS, Jalife J (2015). Ion channel macromolecular complexes in cardiomyocytes: roles in sudden cardiac death. Circ Res.

[34] Bers DM, Perez-Reyes E (1999). Ca channels in cardiac myocytes: structure and function in Ca influx and intracellular Ca release. Cardiovasc Res.

[35] Rahm AK, Lugenbiel P, Schweizer PA, Katus HA, Thomas D (2018). Role of ion channels in heart failure and channelopathies. Biophys Rev.

[36] Harrell MD, Harbi S, Hoffman JF, Zavadil J, Coetzee WA (2007). Large-scale analysis of ion channel gene expression in the mouse heart during perinatal development. Physiol Genomics.

[37] Starnes L, Hall A, Etal D, Cavallo AL, Grabowski P, Gallon J (2024). RYR2 deficient human model identifies calcium handling and metabolic dysfunction impacting pharmacological responses. Front Cardiovasc Med.

[38] Bround MJ, Asghari P, Wambolt RB, Bohunek L, Smits C, Philit M (2012). Cardiac ryanodine receptors control heart rate and rhythmicity in adult mice. Cardiovasc Res.

[39] Guo Y, Cao Y, Jardin BD, Zhang X, Zhou P, Guatimosim S (2023). Ryanodine receptor 2 (RYR2) dysfunction activates the unfolded protein response and perturbs cardiomyocyte maturation. Cardiovasc Res.

[40] Tyser RC, Miranda AM, Chen CM, Davidson SM, Srinivas S, Riley PR (2016). Calcium handling precedes cardiac differentiation to initiate the first heartbeat. Elife.

[41] Bourguignon T, Benoist L, Chadet S, Miquelestorena-Standley E, Fromont G, Ivanes F (2019). Stimulation of murine P2Y11-like purinoreceptor protects against hypoxia/reoxygenation injury and decreases heart graft rejection lesions. J Thorac Cardiovasc Surg.

