# Supplementary material for: The modulation of calcium and chloride channels induces cardiomyocytes from human pluripotent stem cells
Source: Int J Biol Sci. 2025 Jan 1;21(1):95–108. doi: 10.7150/ijbs.95568 (PMC11667823; doi:10.7150/ijbs.95568)
Supplement: Supplementary file 1 — Supplementary figures and tables. [file ijbsv21p0095s1.pdf]

Supplemental Information

The modulation of calcium and chloride channels induces cardiomyocytes from human pluripotent stem cells

Ya Meng[1,2#*], Chunhao Deng[3#], Xia Xiao[2#], Shengnan Wei[2, 4], Chengcheng Song[2, 4], Jiaxian Wang[5], Chon Lok LEI[2], Weiwei Liu[2, 4], Guokai Chen[2, 3, 6*]

1. Guangdong Provincial Key Laboratory of Tumor Interventional Diagnosis and Treatment, Zhuhai People's Hospital, Zhuhai Clinical Medical College of Jinan University, Zhuhai, Guangdong, 519000, China.
2. Faculty of Health Sciences, University of Macau, Taipa, Macau
3. Chinese Medicine and Translational Medicine R&D center, Zhuhai UM Science & Technology Research Institute, Zhuhai, Guangdong, 519031, China
4. Biological Imaging and Stem Cell Core, Faculty of Health Sciences, University of Macau, Taipa, Macau
5. HELP Stem Cell Innovations Co. Ltd., Nanjing, 210000, China
6. MoE Frontiers Science Center for Precision Oncology, University of Macau, Macau SAR, China

Corresponding authors: Ya Meng, yameng@ext.jnu.edu.cn; Guokai Chen, guokaichen@um.edu.mo.

# These authors contributed equally to this paper.

Index

Supplemental Methods

Supplemental Figure legends

Fig. S1 Expression of ion channels in human embryonic heart and dynamic expression of RYR2 during the process of cardiac differentiation.

Fig. S2 Suramin promoted cardiomyocytes derivation from hPSCs.

Fig. S3 Flow cytometry analysis and immunostaining images of TNNT2 after the treatments of ion channel regulators.

Fig. S4 DIDS promoted cardiomyocytes derivation from hPSCs.

Fig. S5 Mechanism study of suramin and DIDS-induced cardiomyocyte differentiation.

Fig. S6 Bioinformatics analysis of gene expression in different treatments during cardiac differentiation.

Fig. S7 Analysis of Multielectrode array (MEA) and expression of subtype cardiomyocyte markers in suramin or DIDS-induced cardiomyocytes.

Supplemental Table

Tab. S1 Key resources table.

Supplemental Figures

Supplemental Methods

Intracellular calcium flux assay

The intracellular calcium flux assay was conducted using Calbryte™ 520 AM (Cat. 20653, AAT Bioquest) according to the manufacturer's protocol. Briefly, H1 cells were treated with CHIR99021 for 24 hours and then cultured in basal medium for an additional day. On day 2, a working solution of 5 μM Calbryte™ 520 AM was added to the cells and incubated at 37 °C in a cell incubator for 60 minutes. After washing with HHBS, fluorescence intensity was measured using Varioskan Lux (ThermoFisher). Subsequently, suramin or DIDS was added to the medium and fluorescence intensity was measured after a 5-minute incubation.

Supplemental Figure legends

Fig. S1 Expression of ion channels in human embryonic heart and dynamic expression of RYR2 during the process of cardiac differentiation.

A Gene expression of ion channels during embryonic heart development at early, middle, and late Carnegie stage (CS). Early, CS 13-14; middle, CS 16-20; late, CS 21-23.

E-F Gene expression of *RYR2* and *NKX2-5* during cardiac differentiation. H1 cells were treated with CHIR99021 (5 μM) for 1 day, and then treated with DMSO (Ctrl), IWP-2 (3 μM) or Suramin (50 μM) from day 2 to day 5, and samples were harvested at day 0 (H1), day 3, day 5, and day 7. Data shown are mean ± SD of three independent experiments. At day 5 and day 7, gene expression of *RYR2* and *NKX2-5* of IWP-2 and Suramin groups showed significant differences compared with control (Ctrl) (*p < 0.05, **p < 0.01).

Fig. S2 Suramin promoted cardiomyocytes derivation from hPSCs.

A Analyses of mRNA expression levels of *NKX2-5* and *TNNT2* at day 10 of cardiac differentiation in H9 hESC. IWP-2 (3 μM) or suramin (50 μM) was added into basal medium from day 2 to day 5.

B Flow cytometry analysis of TNNT2-positive cells in the indicated groups after 12 days of differentiation in H9 hESCs.

C Analyses of mRNA expression levels of *NKX2-5* and *TNNT2* at day 10 of cardiac differentiation in NL4 iPSCs.

D Flow cytometry analysis of TNNT2-positive cells in the indicated groups after 12 days of differentiation in NL4 iPSCs.

E Immunostaining images of H1 before cardiac differentiation. DAPI, blue; NKX2-5, red; TNNT2, green.

Data shown are mean ± SD of three independent experiments (*$p < 0.05$ compared with none-treated control).

Fig. S3 Flow cytometry analysis and immunostaining images of TNNT2 after the treatments of ion channel regulators.

A TNNT2-positive cardiomyocytes were quantified using flow cytometry analysis after 12 days of differentiation.

B Immunostaining images demonstrated the expression levels of TNNT2 in the differentiated cells following 12 days of differentiation. TNNT2, green; DAPI, blue; scale bar, 50 μm.

Fig. S4 DIDS promoted cardiomyocytes derivation from hPSCs.

A Analyses of mRNA expression levels of *NKX2-5* and *TNNT2* at day 10 of cardiac differentiation in H9 hESC. IWP-2 (3 μM) or DIDS (4 μM) was added into basal medium from day 2 to day 5. Data shown are mean ± SD of three independent experiments (*$p < 0.05$ compared with Ctrl).

B Flow cytometry analysis of TNNT2-positive cells in the indicated groups after 12 days of differentiation in H9 hESCs.

C Analyses of mRNA expression levels of *NKX2-5* and *TNNT2* at day 10 of cardiac differentiation in NL4 iPSCs. Data shown are mean ± SD of three independent experiments (*$p < 0.05$ compared with Ctrl).

D Flow cytometry analysis of TNNT2-positive cells in the indicated groups after 12 days of differentiation in NL4 iPSCs.

E-F Cell survival did not exhibit significant differences throughout the process of differentiation. Cell numbers were assessed on days 0, 3, 5, 7, and 9 of differentiation and normalized to the initial cell count at day 0. Black, Ctrl; blue, IWP-2 group; orange, DIDS group; red, suramin group. N=3.

G Gene expression of cardiac markers at day 10 of differentiation. Data shown are mean ± SD of three independent experiments (*$p < 0.05$ compared with IWP-2).

H Immunostaining images represented the sarcomere structure labeled by TNNT2 (green) in Suramin or DIDS-treated cells at day 40 of differentiation. TNNT2, green; DAPI, blue; Scale bar, 50 μm.

Fig. S5 Mechanism study of suramin and DIDS-induced cardiomyocyte differentiation.

A-B Gene expression of *TNNT2* and *NKX2-5* on day 10 of differentiation. Sirti, EX527 10 μM; JNKi, SP600125 3 μM; ERKi, SCH772984 10 μM, P2YRi, P2Y receptor antagonist, AR-C124910XX 3 μM. Data shown are mean ± SD of three independent experiments (*$p < 0.05$ compared with Ctrl).

C Intracellular calcium concentration was enhanced upon administration of suramin and DIDS, as determined using Calbryte™ 590 AM. The calcium concentration was assessed prior to the administration of suramin or DIDS, and subsequently after a 5-minute treatment with these compounds.

D-E Calcium chelator BAPTA downregulated gene expression of cardiac markers *TNNT2* and *NKX2-5*. BAPTA, 50 μM. Data shown are mean ± SD of three independent experiments (*$p < 0.05$ compared with the indicated group).

F-G Gene expression of *NANOG*, *POU5F1* and *RYR2* in H1 and three shRYR2 stable cell lines. shRYR2-2 and shRYR2-3 were used to investigate the role of RYR2 in cardiac differentiation. Data shown are mean ± SD of three independent experiments (*$p < 0.05$ compared with H1).

Fig. S6 Bioinformatics analysis of gene expression in different treatments during cardiac differentiation.

A-C Bubble plot of enriched KEGG pathways from suramin (**A**), DIDS (**B**), and IWR-1 (**C**) up-regulated genes. Rich factor is the ratio of the treatment-regulated gene number to the total gene number of a certain pathway. A Q value is the corrected p value ranging from 0 to 1. The color and size of the dots indicated the range of the Q-value and the number of genes mapped to the certain pathways.

D-F Expression of genes encoded chloride channels (**D**), sodium channels (**E**), potassium channels (**F**) in the indicated groups.

G Gene ontology analysis of suramin, DIDS, or IWR-1 down-regulated genes compared with Ctrl.

Fig. S7 Analysis of Multielectrode array (MEA) and expression of subtype cardiomyocyte markers in suramin or DIDS-induced cardiomyocytes.

A The representative waveform of suramin or DIDS-induced cardiomyocytes was recorded using Axion Biosystems. The suramin or DIDS-induced cardiomyocytes were seeded in the 12-well MEA plates on day 15 of differentiation, and the electrical signals were recorded after 5-10 days.

B The amplitude, beat period and field potential duration (FPD) were measured in suramin or DIDS-induced cardiomyocytes by MEA. Data shown are mean ± SD of 19-30 measurements (*$p < 0.05$).

C-D Gene expression of ventricular marker *IRX4*, and Atrial maker *NPPA* at day 40-50 of differentiation. Data shown are mean ± SD of three independent experiments (*$p < 0.05$ compared with IWP-2 group).

E Immunostaining images represented the level of HCN4 (red) and DAPI (blue) in the differentiated cells at day 40-50 of differentiation. Scale bar, 50 μm.

Supplemental Table

Tab. S1 Key resources table.

REAGENT or RESOURCE	SOURCE	IDENTIFIER	Concentration/ Dilution factor
**Antibody**			
TNNT2 Antibody	DSHB	Cat.# CT3; RRID: AB_528459	1:100
NKX2.5 Antibody	Santa Cruz	Cat.# sc-14033; RRID: AB_650281	1:100
β-Catenin Antibody	Santa Cruz	Cat.# sc-59737; RRID: AB_781850	1:100
MLC2A Antibody	Synaptic Systems	Cat.# 311011; RRID: AB_887737	1:100
HCN4 Antibody	Thermo	Cat.# MA5-45431; RRID: AB_2931885	1:100
Alexa Fluor® 488-AffiniPure Goat Anti-Mouse IgG	Jackson	Cat.# 115-545-071; RRID: AB_2338847	1:100
Alexa Fluor® 594-AffiniPure Donkey Anti-Rabbit IgG	Jackson	Cat.# 711-585-152; RRID: AB_2340621	1:100

**Chemicals, Kits and Recombinant Proteins**			
L-ascorbic acid-2-phosphate magnesium	Sigma	Cat.# A8960	64 µg/mL
Sodium selenium	Sigma	Cat.# S5261	14 ng/mL
holo-transferrin human	Sigma	Cat.# T0665	10 µg/mL
bFGF	From Chen lab (Chen et al., 2012)	N/A	100 ng/mL
Recombinant human TGF-β1	Peprotech	Cat.# 100-21	2 ng/mL
Insulin solution human	Sigma	Cat.# I9278	19.4 µg/mL
Penicillin/Streptomycin	Thermo	Cat.# 15140-122	1:1000
Y-27632	Selleck	Cat.# S1049	5 µM
CHIR-99021	Selleck	Cat.# S2924	5 µM
IWP-2	Selleck	Cat.# S7085	3 µM
Suramin	Tocris	Cat.# 1472	50 µM
4-CMC	Sigma	Cat.# C55402	500 µM
2-APB	Sigma	Cat.# 100065	40 µM
BAPTA	Sigma	Cat.# 196419	10 µM
Dantrolene	Selleck	Cat.# S5478	50 µM
JTV519	Sigma	Cat.# SML0549	10 µM
IP3	Sigma	Cat.# 407137	40 µM
ATP	Sigma	Cat.# A1852	40 µM
3-Deoxyaconitine	MCE	Cat.# HY-N2164	5 µM
Lu AE98134	MCE	Cat.# HY-133910	5 µM
Nav1.1 activator	MCE	Cat.# HY-126429	5 µM
Veratrine	Selleck	Cat.# S3250	3 µM
Bifenthrin	MCE	Cat.# HY-B0824	5 µM
A-803467	Selleck	Cat.# S2785	2.5 µM
Bupivacaine	Selleck	Cat.# S2454	2.5 µM
Dyclonine	Selleck	Cat.# S2041	2.5 µM

Triamterene	Selleck	Cat.# S4080	2.5 µM
Rufinamide	Selleck	Cat.# S1256	2.5 µM
Ambroxol	Selleck	Cat.# S3064	2.5 µM
ICA-27243	Selleck	Cat.# E0105	5 µM
SKA-31	Selleck	Cat.# S0311	5 µM
ML-213	Selleck	Cat.# S6553	5 µM
Retigabine 2HCl	Selleck	Cat.# S4734	5 µM
Repaglinide	Selleck	Cat.# S1426	2.5 µM
Gliquidone	Selleck	Cat.# S3151	2.5 µM
Dofetilide	Selleck	Cat.# S1658	2.5 µM
Tolbutamide	Selleck	Cat.# S2443	2.5 µM
Lubiprostone	Selleck	Cat.# S1675	2.5 µM
DCEBIO	Tocris	Cat.# 1422	2.5 µM
CFTRinh-172	Selleck	Cat.# S7139	2.5 µM
DIDS	Tocris	Cat.# 4523	4 µM
IWR-1	Selleck	Cat.# S7086	2.5 µM
EX527	Selleck	S1541	10 µM
SP600125	Selleck	S1460	3 µM
SCH772984	MCE	HY-50846	10 µM
AR-C124910XX	MCE	HY-110126	3 µM
DAPI	Thermo	Cat.# D1306	0.1 ug/mL
BSA	Sigma	Cat.# A7030	0.5%
Triton™ X-100	Sigma	Cat.# X100	0.1%
Paraformaldehyde	Sigma	Cat.# P6148	4%
Applied Biosystems™ High-Capacity cDNA Reverse Transcription Kit	Applied Biosystems	Cat.# 4368813	
SYBR® Premix Ex Taq™ (Tli RNaseH Plus)	Takara	Cat.# RR420A	
Calbryte™ 520 AM	AAT Bioquest	Cat.# 20653	5 µM

**Software**		
Prism 8	GraphPad Software	https://www.graphpad.com
FlowJo V7.6.1	FlowJo	https://www.flowjo.com
R 3.4.2	R Software	https://www.rproject.org
**Cell Lines**		
Human ESC H1	(Thomson et al., 1998)	N/A
Human ESC H9	(Thomson et al., 1998)	N/A
iPSC NL-4 (NCRM-4)	From NIH	N/A
**Other**		
DMEM/F12	Thermo	Cat.# 11330-032
Matrigel	Corning	Cat.# 354230
TrypLE select enzyme	Thermo	Cat.# 12563-029
RNAiso Plus	Takara	Cat.# 9109
Vectashield Antifade Mounting Medium	Vector	Cat.# H-1000
Chemically Defined Lipid Concentrate	Thermo	Cat.# 11905031
**Primers of Q-PCR**		
**Gene**	**Sequence (5'-3')**	
NKX2.5-Reverse	CAGCTCTTTCTTTTCGGCTCTA	
NKX2-5-Forward	CAAGTGTGCGTCTGCCTTT	
RYR2-Forward	ACAACAGAAGCTATGCTTGGC	
RYR2-Reverse	GAGGAGTGTTCGATGACCACC	
TBP-Forward	TGAGTTGCTCATACCGTGCTGCTA	
TBP-Reverse	CCCTCAAACCAACTTGTCAACAGC	
TNNT2-Forward	GGAGGAGTCCAAACCAAAGCC	
TNNT2-Reverse	TCAAAGTCCACTCTCTCTCCATC	
ITPR2-Forward	CACCTTGGGGTTAGTGGATGA	
ITPR2-Reverse	CTCGGTGTGGTTCCCTTGT	
ITPR3-Forward	CCAAGCAGACTAAGCAGGACA	

ITPR3-Reverse	ACACTGCCATACTTCACGACA
RYR1-Forward	CTCCGCCTCTTTCATGGACAT
RYR1-Reverse	CTGCCCGGTAGTGACATGC
RYR3-Forward	GGACTTGGGAATCGCCTGTG
RYR3-Reverse	GCTCTGACAGATAGGGACTGTTC
WNT2-Forward	AAAGAAGATGGGAAGCGCCA
WNT2-Reverse	TTCATCAGGGCTCTGGCATC
WNT3-Forward	CGCACGACTATCCTGGAC
WNT3-Reverse	GAGGCGCTGTCATACTTGTC
WNT5A-Forward	GCCCAGGTTGTAATTGAAGC
WNT5A-Reverse	TGGCACAGTTTCTTCTGTCC
NANOG-Forward	GATGCCTCACACGGAGACTG
NANOG-Reverse	GCAGAAGTGGGTTGTTTGCC
POU5F1-Forward	AACCTGGAGTTTGTGCCAGGGTTT
POU5F1-Reverse	TGAACTTCACCTTCCCTCCAACCA
MYL2-Forward	TGTCCCTACCTTGTCTGTTAGCCA
MYL2-Reverse	ATTGGAACATGGCCTCTGGATGGA
MYL7-Forward	ACATCATCACCCATGGAGACGAGA
MYL7-Reverse	GCAACAGAGTTTATTGAGGTGCCC
HCN4-Forward	GACTGCTGGGTGTCCATCAA
HCN4-Reverse	AGAGCGCGTAGGAGTACTGCTT
IRX4-Forward	TTGGACTCCTGGGAACATGGACAA
IRX4-Reverse	ATGCTTCAGGGTATCTGGCCTCTT
NPPA-Forward	GGGTCTCTGCTGCATTTGTGTCAT
NPPA-Reverse	AGAGGCGAGGAAGTCACCATCAAA
CACNA1D-Forward	GACCAACTTCTCAGCCGAATA
CACNA1D- Reverse	GTGCAGAGGTGCTCATAGTT
TNNI1-Forward	CCGGAAGTCGAGAGAAAACCC
TNNI1- Reverse	TGGCCTCAATGTCGTATCGC

Figure. S1

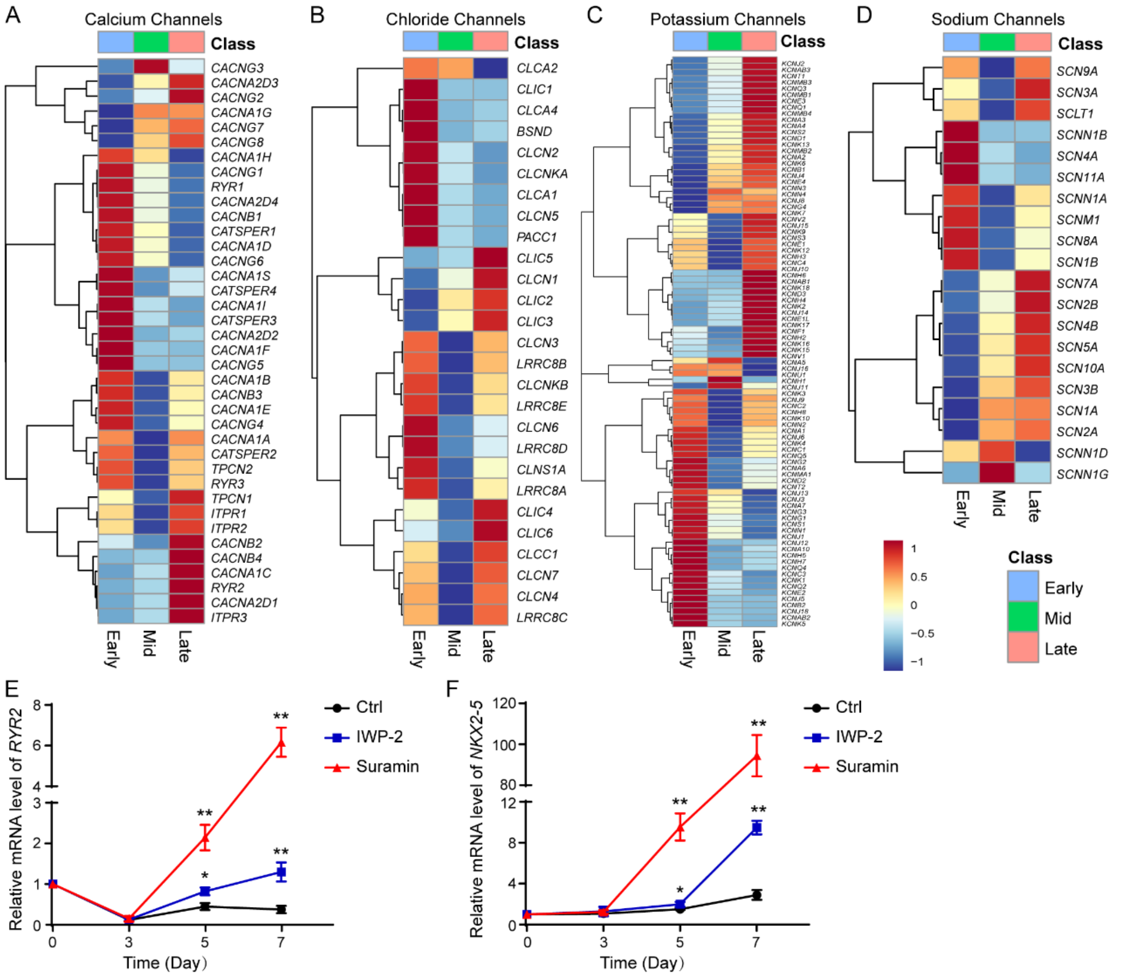
A
Calcium Channels
Class
CACNG3
CACNA2D3
CACNG2
CACNA1G
CACNG7
CACNG8
CACNA1H
CACNG1
RYR1
CACNA2D4
CACNB1
CATSPER1
CACNA1D
CACNG6
CACNA1S
CATSPER4
CACNA1I
CATSPER3
CACNA2D2
CACNA1F
CACNG5
CACNA1B
CACNB3
CACNA1E
CACNG4
CACNA1A
CATSPER2
TPCN2
RYR3
TPCN1
ITPR1
ITPR2
CACNB2
CACNB4
CACNA1C
RYR2
CACNA2D1
ITPR3
Early
Mid
Late
B
Chloride Channels
Class
CLCA2
CLIC1
CLCA4
BSND
CLCN2
CLCNKA
CLCA1
CLCN5
PACC1
CLIC5
CLCN1
CLIC2
CLIC3
CLCN3
LRRC8B
CLCNKB
LRRC8E
CLCN6
LRRC8D
CLNS1A
LRRC8A
CLIC4
CLIC6
CLCC1
CLCN7
CLCN4
LRRC8C
Early
Mid
Late
C
Potassium Channels
Class
Early
Mid
Late
D
Sodium Channels
Class
SCN9A
SCN3A
SCLT1
SCNN1B
SCN4A
SCN11A
SCNN1A
SCNM1
SCN8A
SCN1B
SCN7A
SCN2B
SCN4B
SCN5A
SCN10A
SCN3B
SCN1A
SCN2A
SCNN1D
SCNN1G
Early
Mid
Late
1
0.5
0
−0.5
−1
Class
Early
Mid
Late
E
Relative mRNA level of RYR2
Time (Day)
Ctrl
IWP-2
Suramin
F
Relative mRNA level of NKX2-5
Time (Day)
Ctrl
IWP-2
Suramin

Figure. S2

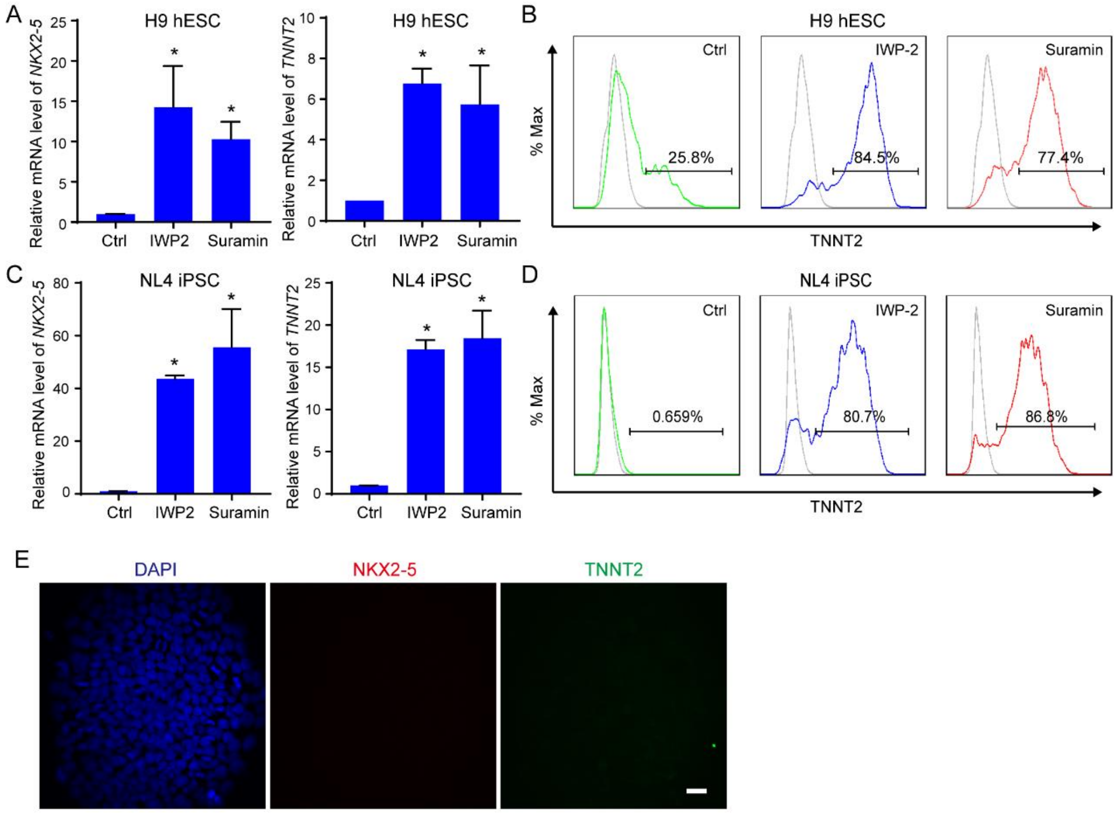
A
H9 hESC
Relative mRNA level of NKX2-5
Relative mRNA level of TNNT2
Ctrl
IWP2
Suramin
B
H9 hESC
% Max
TNNT2
Ctrl
IWP-2
Suramin
25.8%
84.5%
77.4%
C
NL4 iPSC
D
NL4 iPSC
0.659%
80.7%
86.8%
E
DAPI
NKX2-5
TNNT2

Figure. S3

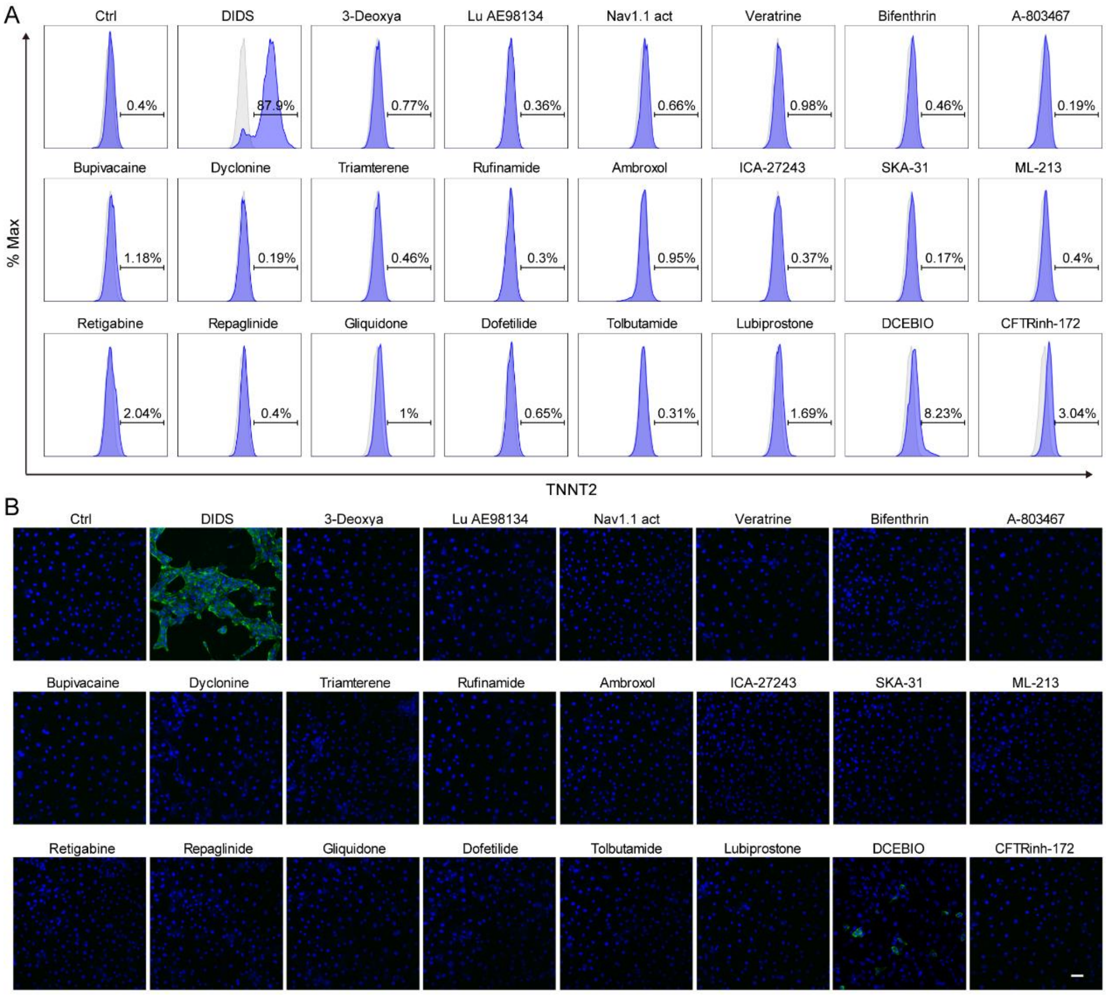
A
Ctrl
0.4%
DIDS
87.9%
3-Deoxya
0.77%
Lu AE98134
0.36%
Nav1.1 act
0.66%
Veratrine
0.98%
Bifenthrin
0.46%
A-803467
0.19%
Bupivacaine
1.18%
Dyclonine
0.19%
Triamterene
0.46%
Rufinamide
0.3%
Ambroxol
0.95%
ICA-27243
0.37%
SKA-31
0.17%
ML-213
0.4%
Retigabine
2.04%
Repaglinide
0.4%
Gliquidone
1%
Dofetilide
0.65%
Tolbutamide
0.31%
Lubiprostone
1.69%
DCEBIO
8.23%
CFTRinh-172
3.04%
% Max
TNNT2
B
Ctrl
DIDS
3-Deoxya
Lu AE98134
Nav1.1 act
Veratrine
Bifenthrin
A-803467
Bupivacaine
Dyclonine
Triamterene
Rufinamide
Ambroxol
ICA-27243
SKA-31
ML-213
Retigabine
Repaglinide
Gliquidone
Dofetilide
Tolbutamide
Lubiprostone
DCEBIO
CFTRinh-172

Figure. S4

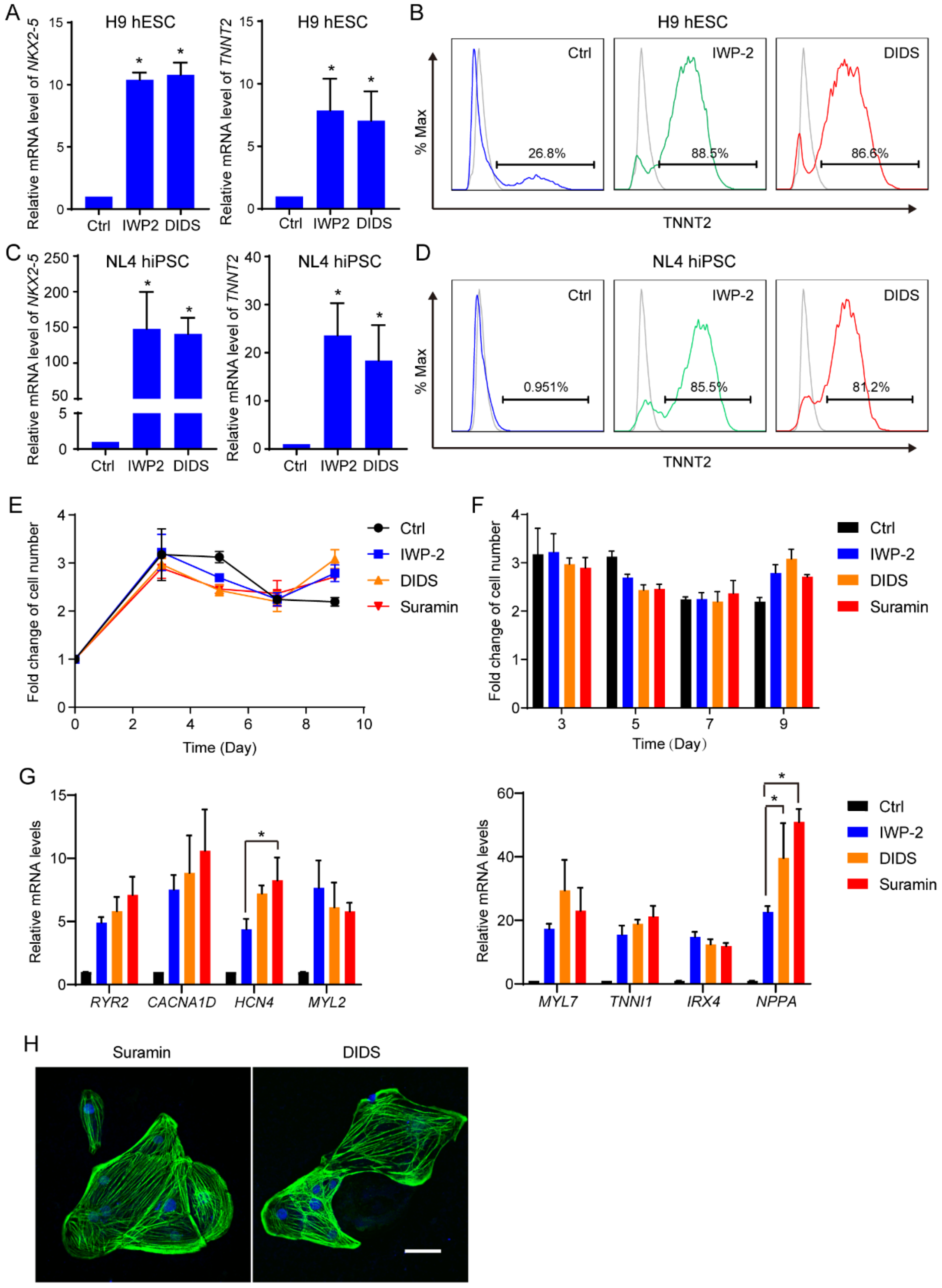

A
H9 hESC
Relative mRNA level of NKX2-5
Ctrl IWP2 DIDS
H9 hESC
Relative mRNA level of TNNT2
B
H9 hESC
Ctrl 26.8%
IWP-2 88.5%
DIDS 86.6%
% Max
TNNT2
C
NL4 hiPSC
Relative mRNA level of NKX2-5
NL4 hiPSC
Relative mRNA level of TNNT2
D
NL4 hiPSC
Ctrl 0.951%
IWP-2 85.5%
DIDS 81.2%
% Max
TNNT2
E
Fold change of cell number
Time (Day)
Ctrl
IWP-2
DIDS
Suramin
F
Fold change of cell number
Time (Day)
G
Relative mRNA levels
RYR2 CACNA1D HCN4 MYL2
MYL7 TNNI1 IRX4 NPPA
H
Suramin
DIDS

Figure. S5

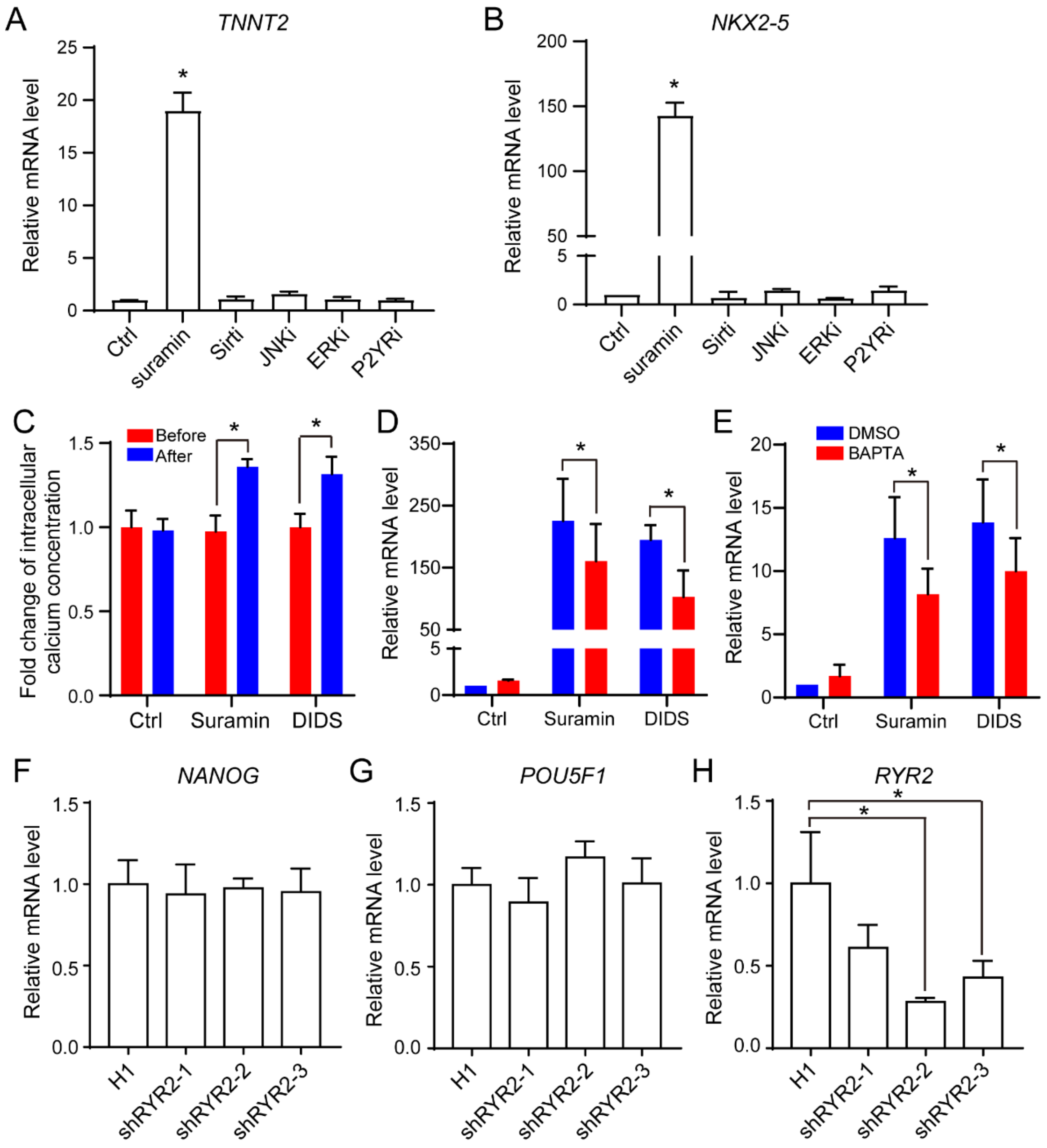
A
TNNT2
Relative mRNA level
25
20
15
10
5
0
*
Ctrl
suramin
Sirti
JNKi
ERKi
P2YRi
B
NKX2-5
Relative mRNA level
200
150
100
50
5
0
*
Ctrl
suramin
Sirti
JNKi
ERKi
P2YRi
C
Before
After
Fold change of intracellular calcium concentration
1.5
1.0
0.5
0.0
*
*
Ctrl
Suramin
DIDS
D
Relative mRNA level
350
250
150
50
5
0
*
*
Ctrl
Suramin
DIDS
E
DMSO
BAPTA
Relative mRNA level
20
15
10
5
0
*
*
Ctrl
Suramin
DIDS
F
NANOG
Relative mRNA level
1.5
1.0
0.5
0.0
H1
shRYR2-1
shRYR2-2
shRYR2-3
G
POU5F1
Relative mRNA level
1.5
1.0
0.5
0.0
H1
shRYR2-1
shRYR2-2
shRYR2-3
H
RYR2
Relative mRNA level
1.5
1.0
0.5
0.0
*
*
H1
shRYR2-1
shRYR2-2
shRYR2-3

Figure. S6

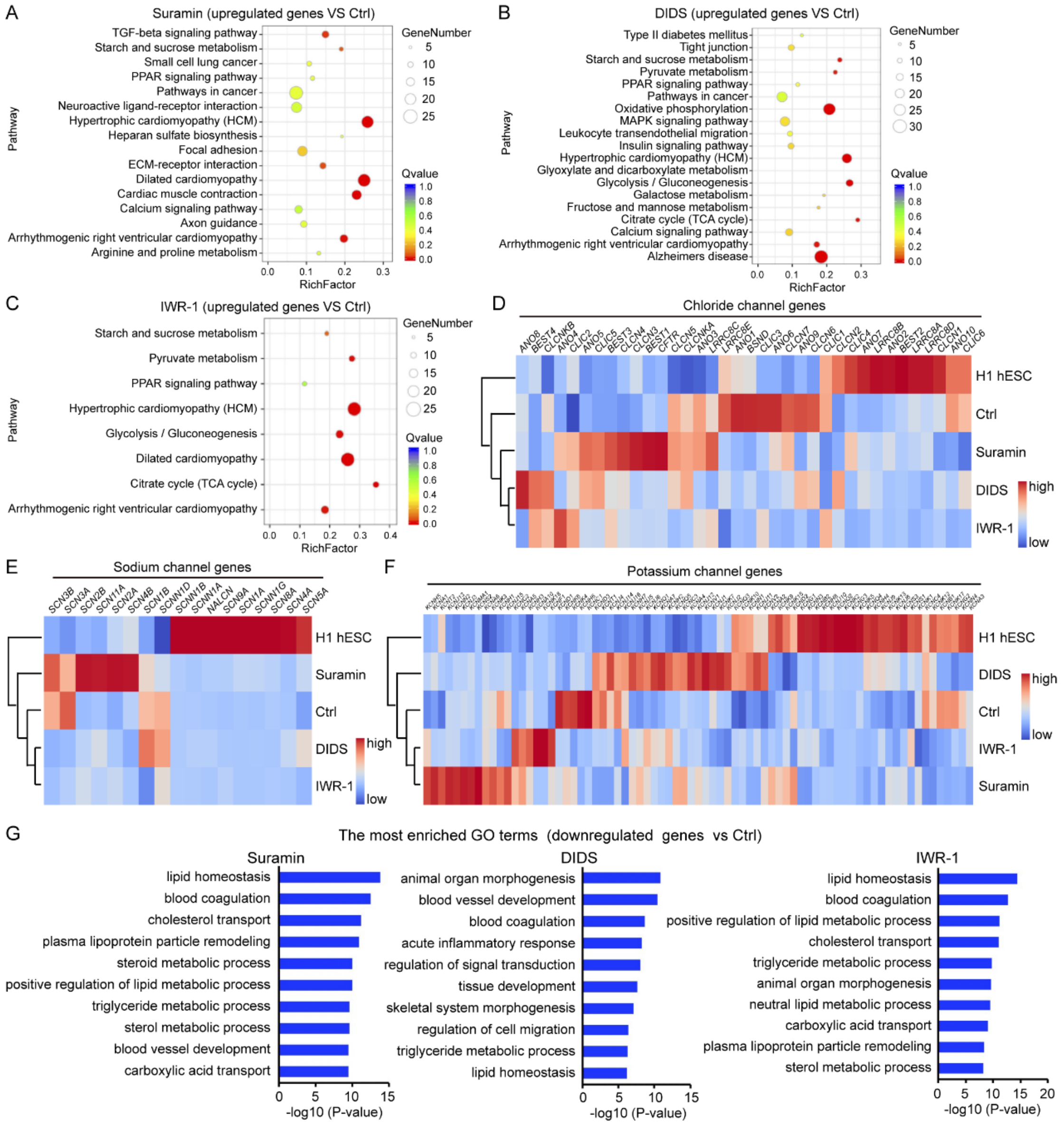

A
Suramin (upregulated genes VS Ctrl)
TGF-beta signaling pathway
Starch and sucrose metabolism
Small cell lung cancer
PPAR signaling pathway
Pathways in cancer
Neuroactive ligand-receptor interaction
Hypertrophic cardiomyopathy (HCM)
Heparan sulfate biosynthesis
Focal adhesion
ECM-receptor interaction
Dilated cardiomyopathy
Cardiac muscle contraction
Calcium signaling pathway
Axon guidance
Arrhythmogenic right ventricular cardiomyopathy
Arginine and proline metabolism
Pathway
RichFactor
GeneNumber
Qvalue
B
DIDS (upregulated genes VS Ctrl)
Type II diabetes mellitus
Tight junction
Starch and sucrose metabolism
Pyruvate metabolism
PPAR signaling pathway
Pathways in cancer
Oxidative phosphorylation
MAPK signaling pathway
Leukocyte transendothelial migration
Insulin signaling pathway
Hypertrophic cardiomyopathy (HCM)
Glyoxylate and dicarboxylate metabolism
Glycolysis / Gluconeogenesis
Galactose metabolism
Fructose and mannose metabolism
Citrate cycle (TCA cycle)
Calcium signaling pathway
Arrhythmogenic right ventricular cardiomyopathy
Alzheimers disease
Pathway
RichFactor
GeneNumber
Qvalue
C
IWR-1 (upregulated genes VS Ctrl)
Starch and sucrose metabolism
Pyruvate metabolism
PPAR signaling pathway
Hypertrophic cardiomyopathy (HCM)
Glycolysis / Gluconeogenesis
Dilated cardiomyopathy
Citrate cycle (TCA cycle)
Arrhythmogenic right ventricular cardiomyopathy
Pathway
RichFactor
GeneNumber
Qvalue
D
Chloride channel genes
H1 hESC
Ctrl
Suramin
DIDS
IWR-1
high
low
E
Sodium channel genes
SCN3B SCN3A SCN2B SCN11A SCN2A SCN4B SCN1B SCNN1D SCNN1B SCNN1A NALCN SCN9A SCN1A SCNN1G SCN8A SCN4A SCN5A
H1 hESC
Suramin
Ctrl
DIDS
IWR-1
high
low
F
Potassium channel genes
H1 hESC
DIDS
Ctrl
IWR-1
Suramin
high
low
G
The most enriched GO terms (downregulated genes vs Ctrl)
Suramin
lipid homeostasis
blood coagulation
cholesterol transport
plasma lipoprotein particle remodeling
steroid metabolic process
positive regulation of lipid metabolic process
triglyceride metabolic process
sterol metabolic process
blood vessel development
carboxylic acid transport
-log10 (P-value)
DIDS
animal organ morphogenesis
blood vessel development
blood coagulation
acute inflammatory response
regulation of signal transduction
tissue development
skeletal system morphogenesis
regulation of cell migration
triglyceride metabolic process
lipid homeostasis
-log10 (P-value)
IWR-1
lipid homeostasis
blood coagulation
positive regulation of lipid metabolic process
cholesterol transport
triglyceride metabolic process
animal organ morphogenesis
neutral lipid metabolic process
carboxylic acid transport
plasma lipoprotein particle remodeling
sterol metabolic process
-log10 (P-value)

Figure. S7

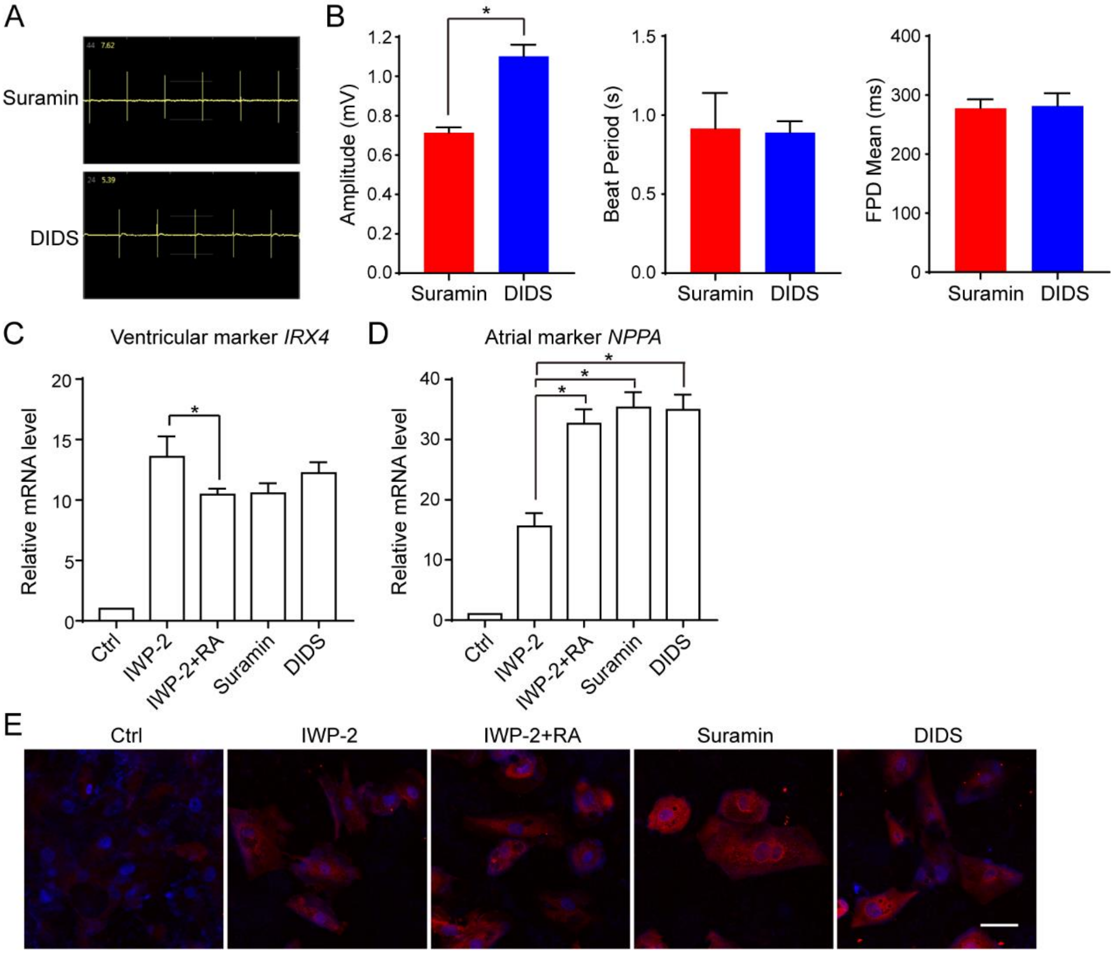
A
Suramin
DIDS
B
Amplitude (mV)
Suramin
DIDS
*
Beat Period (s)
FPD Mean (ms)
C
Ventricular marker IRX4
Relative mRNA level
Ctrl
IWP-2
IWP-2+RA
Suramin
DIDS
D
Atrial marker NPPA
E
Ctrl
IWP-2
IWP-2+RA
Suramin
DIDS
